# A *Drosophila* model of insulin resistance associated with the human TRIB3 Q/R polymorphism

**DOI:** 10.1242/dmm.030619

**Published:** 2017-12-01

**Authors:** Zachary Fischer, Rahul Das, Anna Shipman, Jin-Yuan Fan, Laramie Pence, Samuel Bouyain, Leonard L. Dobens

**Affiliations:** Division of Molecular Biology and Biochemistry, School of Biological Sciences, University of Missouri-Kansas City, Kansas City, MO 64110, USA

**Keywords:** Tribbles, Akt, Insulin signaling, *Drosophila*

## Abstract

Members of the Tribbles family of proteins are conserved pseudokinases with diverse roles in cell growth and proliferation. Both *Drosophila* Tribbles (Trbl) and vertebrate Trib3 proteins bind to the kinase Akt (Akt1) to block its phosphorylation activation and reduce downstream insulin-stimulated anabolism. A single nucleotide polymorphism (SNP) variant in human TRIB3, which results in a glutamine (Q) to arginine (R) missense mutation in a conserved motif at position 84, confers stronger Akt binding, resulting in reduced Akt phosphorylation, and is associated with a predisposition to Type 2 diabetes, cardiovascular disease, diabetic nephropathy, chronic kidney disease and leukemogenesis. Here, we used a *Drosophila* model to understand the importance of the conserved R residue in several Trbl functions. In the fly fat body, misexpression of a site-directed Q mutation at position R141 resulted in weakened binding to *Drosophila* Akt (dAkt), leading to increased levels of phospho-dAkt, increased cell and tissue size, and increases in the levels of stored glycogen and triglycerides. Consistent with the functional conservation of this arginine in modulating Akt activity, mouse Trib3 R84 misexpressed in the fly fat body blocked dAkt phosphorylation with a strength similar to wild-type Trbl. Limited mutational analysis shows that the R141 site dictates the strength of Akt binding but does not affect other Trbl-dependent developmental processes, suggesting a specificity that could serve as a drug target for metabolic diseases.

## Abstract (precis)

**Summary:** The insulin signaling inhibitor tribbles 3 (TRIB3) variant associated with Type II diabetes has parallel effects in a fly model system on Tribbles-regulated insulin signaling, growth and Akt activation.

## INTRODUCTION

Genetics heavily influence susceptibility to insulin resistance syndrome (including Type 2 diabetes) (Bouret et al., 2015; Eckardt et al., 2011; Frayling et al., 2003; Kahn et al., 2014; Mitchell et al., 1994; Weijnen et al., 2002). Genome-wide association studies (GWAS) have identified >90 loci associated with diabetic phenotypes, including genes associated with insulin production or secretion (Dimas et al., 2014; Frayling, 2007; Ghosh et al., 2000; Permutt et al., 2001; Renström et al., 2009). However, GWAS approaches have identified only a few genes that function in insulin responsive cells, a result that could be caused by poor coverage of genetic markers, environmental influences or problems classifying multifactorial diseases (Prudente et al., 2012a; Visscher et al., 2012; Voight et al., 2010; Zeggini et al., 2008).

A primary cause of insulin resistance in responsive tissue is an alteration in the activity of the insulin receptor or key downstream pathway mediators. The binding of insulin and insulin-like peptides to cognate cell surface insulin receptors triggers a phosphorylation cascade that results in activation of the kinase Akt (Akt1), which in turn phosphorylates several targets, including: (1) the Rheb-specific GTPase-activating protein (GAP) Tsc2 (Gig), to induce Target of rapamycin complex 1 (TORC1; or Tor)-mediated protein and lipid biosynthesis (Garami et al., 2003; Miron et al., 2003; Montagne et al., 1999; Porstmann et al., 2008); (2) GSK-3β (Sgg), to inhibit gluconeogenesis and boost anabolic gene expression by stabilizing Myc (Parisi et al., 2011; Teleman et al., 2008); (3) the pro-apoptotic protein BAD, to inhibit apoptosis (Datta et al., 1997); and (4) the transcription factor Foxo, to block its nuclear localization and reduce expression of catabolic and apoptotic Foxo target genes (Jünger et al., 2003). Given the central, but complex, role of Akt in mediating insulin-regulated cell growth and survival, it is not surprising that aberrant alteration of Akt activity underlies diabetic metabolic disease (reviewed in Zdychová and Komers, 2005).

Several endogenous mechanisms feedback to attenuate the strength of the insulin response, including negative feedback from phosphatases and target genes that bind and modulate the activity of key pathway components (Kockel et al., 2010; Poltilove et al., 2000). Among the latter is TRIB3, a member of the Tribbles family of pseudokinase adaptor proteins, which increases in levels following starvation and exercise, and binds AKT to block its activation and effectively reduce tissue growth and increase circulating levels of glucose (Du et al., 2003; Lima et al., 2009; Schwarzer et al., 2006). Overexpression of Trib3 decreases Akt protein activation in the liver and muscle, whereas knockdown of Trib3 expression increases Akt in these tissues (Hegedus et al., 2007; Koo et al., 2004; Matos et al., 2010; Matsushima et al., 2006; Wang et al., 2009). Trib3 might further regulate metabolism in diverse tissues via poorly understood interactions with (1) acetyl-coenzyme A carboxylase (ACC) in the liver to regulate lipid metabolism; (2) Mtor and Rictor to regulate growth in renal tubular cells (Borsting et al., 2014); and (3) ATF4 in β-cells to regulate insulin production (Liew et al., 2010; Qi et al., 2006). In humans, TRIB3 levels are aberrantly increased in both insulin resistant adults and obese adults (Liu et al., 2012; Oberkofler et al., 2010). Trib3 knockdown in rat and mouse diabetic models reduces the destructive effects of diet on cardiomyopathy, making Trib3 a candidate target to alleviate metabolic disease (Ti et al., 2011; Wang et al., 2012).

A prevalent single nucleotide polymorphism (dsSNP ID: rs2295490) in the human TRIB3 coding sequence predisposes carriers to early onset insulin resistance and early onset Type 2 diabetes (Andreozzi et al., 2008; De Cosmo et al., 2007; Formoso et al., 2011; Gong et al., 2009; Prudente et al., 2005, 2010, 2013; Shi et al., 2009; Zhang et al., 2015; reviewed in Hegedus et al., 2006; Prudente et al., 2015). The allele frequency ranges from 13% in European and African individuals to 25-27% in Japanese and Chinese individuals, and has been connected to glucose homeostasis, metabolic syndrome, atherosclerosis, cardiovascular disease, diabetic nephropathy and chronic kidney disease (Prudente et al., 2012b).

The TRIB3 SNP associated with insulin resistance results in a missense mutation, changing a polar amino acid glutamine (Q) at position 84 in the conserved kinase-like domain to basic arginine (R). In TRIB1 and TRIB2, the position is occupied invariably by an arginine and an R/L mutation in TRIB1 in this motif (R107L) associated with Down syndrome-related acute megakaryocytic leukemia (AML) enhances ERK (MAPK) protein phosphorylation and C/EBPα (CEBPA) degradation, pointing to its central function in target regulation (Yokoyama et al., 2012). Misexpression of the TRIB3 R84 variant in human hepatoma cell lines blocks AKT phosphorylation at Ser473 more effectively than the Q variant, leading to the notion that the R variant is a more potent inhibitor of AKT activation, and, consistent with this, the R variant physically binds AKT more strongly than the Q variant (Andreozzi et al., 2008; Gong et al., 2009; Prudente et al., 2005, 2010). Compared with Q84, R84 more efficiently impairs insulin secretion in β-cells (Liew et al., 2010), and decreases nitric oxide release and increases MAPK protein activity in endothelial cells, early steps in atherosclerosis (Formoso et al., 2011). Consistent with the idea that the R84 variant is a gain-of-function allele, insulin-mediated glucose disposal was progressively impaired in endothelial cells from QQ to QR to RR patients (Andreozzi et al., 2008).

Tribbles proteins were first identified in *Drosophila*, and Tribbles family members throughout the metazoan lineage share a conserved function to bind key regulatory proteins and direct their proteasomal degradation to modulate multiple signaling pathways (Dobens and Bouyain, 2012). In *Drosophila*, a conserved insulin signaling pathway links cellular nutrient homeostasis to the control of diverse developmental processes, including body size and sexual maturation (Koyama et al., 2013). We identified *Drosophila* Tribbles (Trbl) as a strong inhibitor of Akt-mediated effects on tissue growth, fat mobilization, metabolite storage and the timing of key life-cycle events dependent on these functions, including pupariation and eclosion, indicating that the functions of the Tribbles family members are evolutionarily conserved (Das et al., 2014). Tribbles family proteins share an extensive sequence homology in the central kinase-like domain, including a motif that spans the R/Q residue associated with metabolic disease in humans, so we sought to test if this site is required for Trbl-regulated functions in flies. We show here that, like its human counterpart, the R variant in flies is a more potent binder and inhibitor of Akt-mediated insulin signaling than an engineered Q variant. Mutations at the R141 site are specific for Akt-mediated signaling and do not compromise the potent inhibitory effect of Trbl on developmental processes regulated by the Trbl targets C/EBP (Slbo) and Cdc25 phosphatase (String). A mouse Trib3 R variant robustly blocks Akt activation in the fly model, consistent with the conserved function of this site. Together, these data imply that the human Q84 variant, which appears only late in the course of human evolution, might confer protection from Type 2 diabetes brought on by modern diets. Because mutations tested at R141 have no effects on other Trbl-dependent developmental events, this site could serve as a target for small molecule inhibitors specifically to improve insulin sensitivity in target tissues.

## RESULTS

### The R141Q mutation reduces Trbl-mediated inhibition of Akt and cell growth

**Fig. 1.**
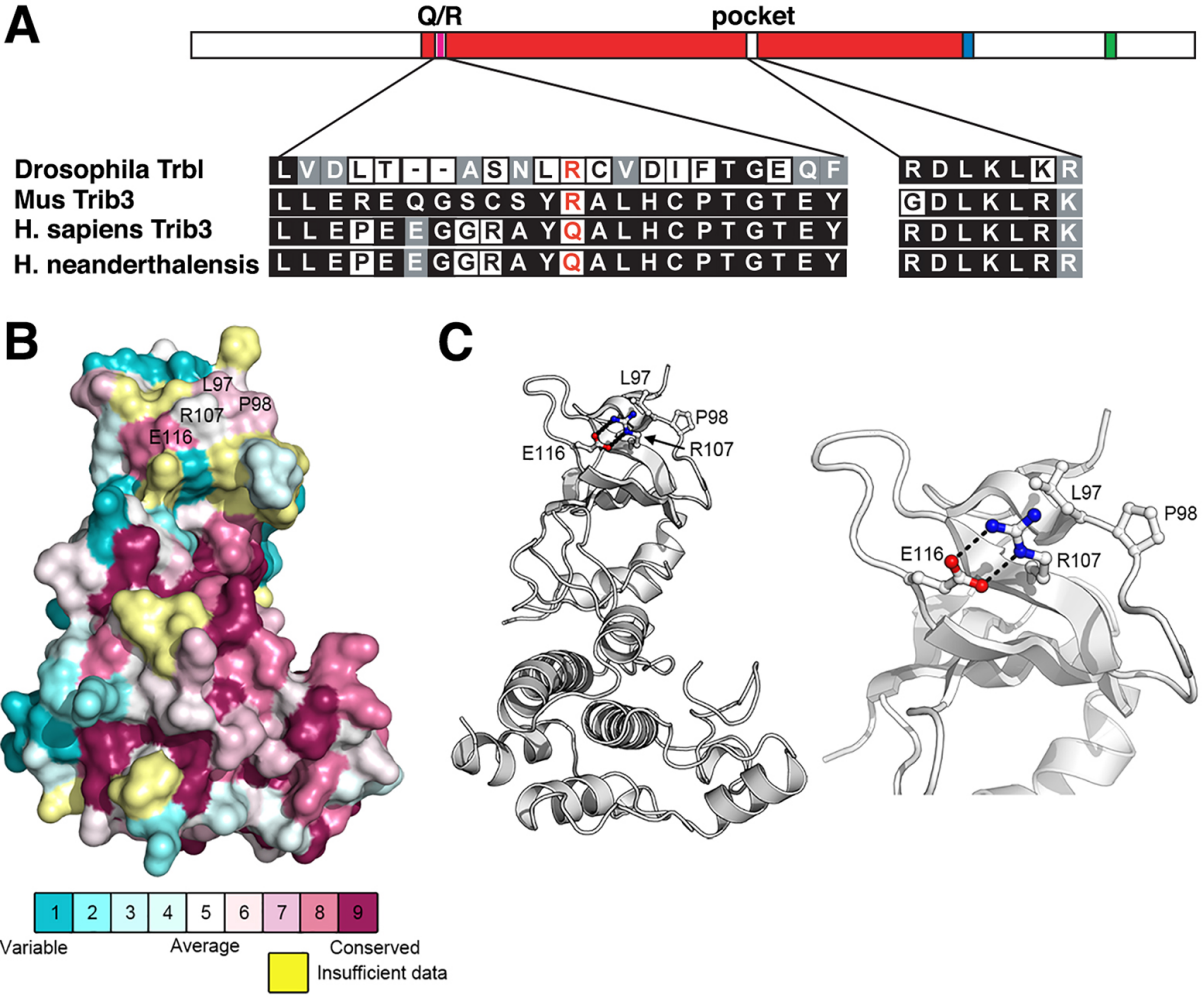
**The human TRIB3 84 position is highly conserved and contains R at this site in most Trib proteins.** (A) Amino acid sequence alignment of key domains in *Drosophila* Trbl and mouse, human and Neanderthal Trib3 proteins. The site corresponding to human TRIB3 Q84 is highlighted in red. (B) Surface mapping of conserved and variable residues in Tribbles and its TRIB1-3 homologs. Residue conservation was assessed using ConSurf (Ashkenazy et al., 2016), and residues are colored according to their conservation from blue (variable) to dark magenta (conserved) on a surface representation of the human TRIB1 kinase-like domain (PDB ID 5CEM) (Murphy et al., 2015). Residues for which data were insufficient to calculate a conservation score are colored yellow. (C) Left panel: a ribbon diagram of human TRIB1 showing conserved residues surrounding R107 (Q84 in human TRIB3). Black dashed lines denote potential salt bridge interactions between R107 and E116 (Andreozzi et al., 2008). A close-up view of the site is shown on the right panel.

We aligned the amino acid sequence of fly Trbl with human TRIB3 and identified a well-conserved region from 136-165 in Trbl that corresponds to a region from 80-109 in TRIB3 and is located N-terminal to the well-conserved central domain of the proteins (Fig. 1A; Fig. S1A,B). The homology in this domain includes an R141 arginine residue in fly Trbl that corresponds to the Q/R84 variant site in human TRIB3. Interestingly, the predominant Q84 glutamine variant is unique to the genus Homo, as the rare R variant associated with predisposition to human Type 2 diabetes is found at this position in Trib3 in other vertebrates and in Trib1 and Trib2 in all vertebrates. Examining this motif in Tribbles family members throughout the metazoan lineage, an R is favored at this position almost universally, including for *Drosophila* Trbl, the focus here (Fig. 1A; Fig. S1C).

The Trib3 SNP associated with insulin resistance results is a missense mutation, resulting in a change from a polar amino acid residue glutamine (Q) at position 84 in the conserved kinase-like domain to a basic one (R). To gain more insight into the possible role of this region in Tribbles, we analyzed the conservation of surface residues to identify potential protein-binding sites (Fig. 1B). We constructed a multiple sequence alignment using sequences of Tribbles, as well as its homologs Trib1-3, and evaluated the conservation of surface residues with the program ConSurf (Ashkenazy et al., 2016). In the human TRIB1 crystal structure (Murphy et al., 2015) this amino acid (R107) lies within a moderately conserved site in the N-terminal lobe of the conserved kinase-like domain and mediates an intramolecular salt bridge interaction with a conserved glutamate residue (E116 in human TRIB1, Fig. 1C) (Andreozzi et al., 2008). Although changing the arginine for a glutamine would disrupt this interaction, the glutamine side chain would still be expected to form a hydrogen bond with E116 so that there would be minimal structural disruption in this region. As such, it seems more likely that the consequence of the mutation is to alter the binding of Trib3 to a currently unknown protein at this site and an attractive possibility is Akt, which we have shown is a target of Tribbles in fly tissue. To test the effect of the Q variant in the fly model system, we designed a mutant Trbl (R141Q) and compared its activity with wild-type (WT) Trbl in insulin-sensitive tissues.

**Fig. 2.**
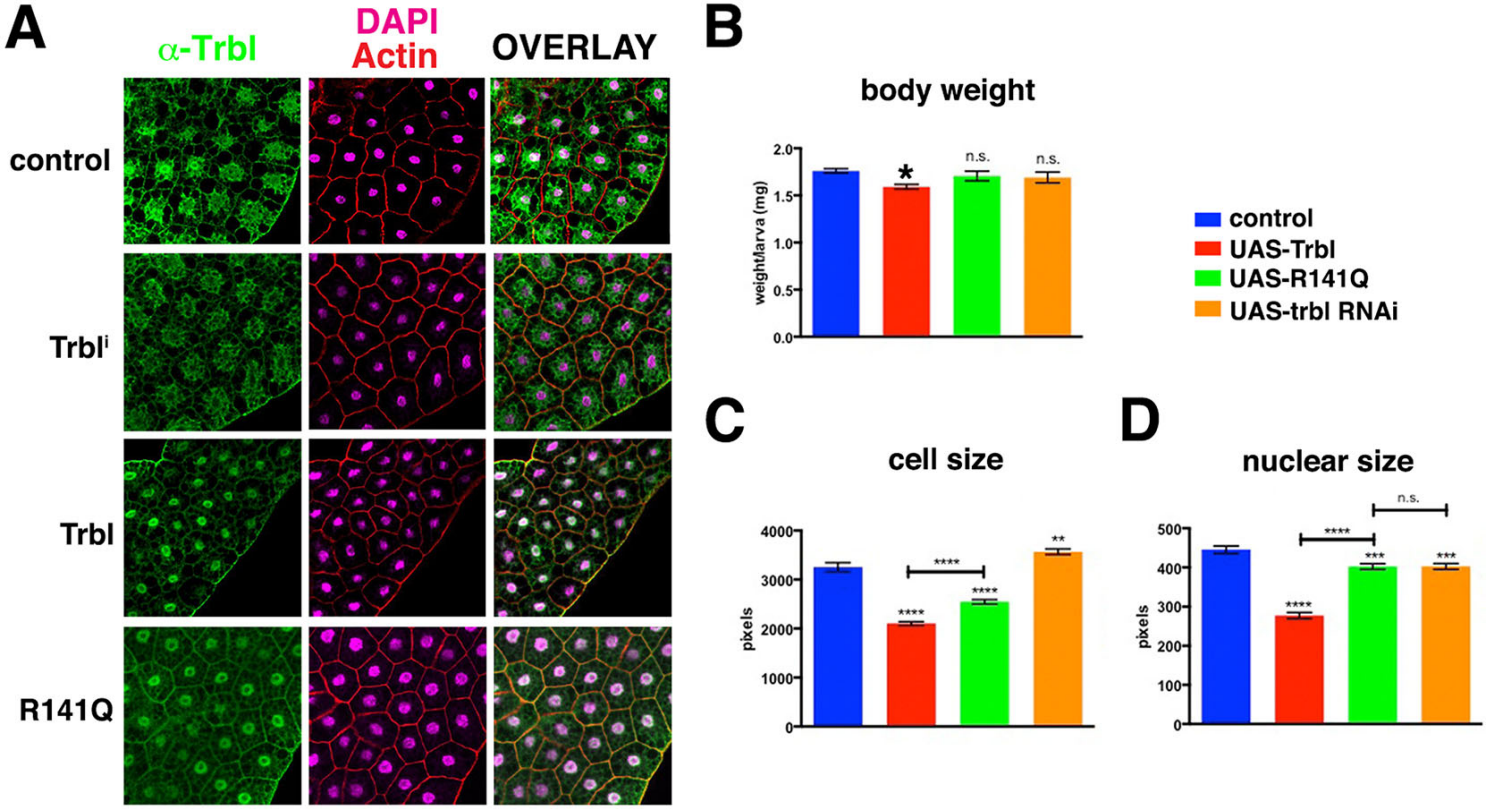
**Fat body misexpression of R141Q: subcellular distribution and effect on cell size.** (A) Optical cross-section of fat body expressing *UAS-lacZ* (control), *trbl* RNAi (Trbl^i^), Trbl or R141Q and immunostained for Trbl protein levels and distribution using specific antisera; DAPI/actin counterstains highlight cell size. Endogenous Trbl protein is nuclear, weakly cytoplasmic and strongly cortical as shown previously, and *trbl* RNAi reduces the strength of antisera staining. Trbl misexpression increases levels in the cell and the subcellular distribution of Trbl is unchanged by the R141Q mutation. Genotypes: (1) control, *r4-GAL4/+*; (2) Trbl^i^, *P{GD11640}v22114/r4-GAL4*; (3) Trbl, *UAS-trbl/+; r4-GAL4/+*; (4) R141Q, *UAS-trbl^R141Q^/+; r4-GAL4/+*. Note that all images were taken with identical confocal parameters so that the strength of staining is comparable. (B) Larval body weight analysis shows that misexpression of R141Q reduces body weight less than WT Trbl. By contrast, weight differences between control and R141Q were not significant (n.s.). *n*=20. (C) Misexpression of R141Q in fat body of age-matched larvae reduced fat-body cell size less significantly than WT Trbl. *n*=100. (D) Misexpression of R141Q fat body of age-matched larvae reduced fat-body nuclear size less significantly than WT Trbl. *n*=100. Statistical analysis was performed with one-way ANOVA followed by Tukey post hoc using GraphPad Prism. Data are mean±s.e.m.

During the active feeding stage of *Drosophila* larval development, the fat body (equivalent to mammalian adipose and liver tissue) stores nutrients in the form of lipid droplets to supply the animal with essential nutrients during periods of starvation or during pupal stages when feeding ceases (Butterworth et al., 1965). The fat body is highly sensitive to insulin signaling with systemic effect on larval growth and maturation (Britton et al., 2002). In fat body cells, specific antisera detect endogenous Trbl localized to the cytoplasm, cell cortex and nucleus (Fig. 2A, top row) and misexpression of a *UAS-trbl^i^* RNAi transgene using the fat body-specific *r4-GAL4* driver (Lee and Park, 2004) reduced levels of Trbl staining in these subcellular regions (Fig. 2A), whereas misexpression of a *UAS-trbl* transgene resulted in more prominent nuclear and membrane localization of the Trbl signal (Fig. 2A). Misexpression of *UAS-trbl^R141Q^* under similar conditions resulted in an identical subcellular Trbl distribution (Fig. 2A, bottom row), suggesting that the R141Q mutation does not strongly affect intracellular localization of Trbl.

As shown previously (Das et al., 2014), misexpression of WT Trbl in the fat body decreased larval body weight and cell size significantly compared with misexpression of a *lacZ* transgene as a control (Fig. 2B). By contrast, *UAS- trbl^R141Q^* misexpression led to no significant reduction in larval body weight compared with the control (Fig. 2B). Consistent with its effects on body weight, Trbl^R141Q^ misexpression led to a smaller reduction in cell and nuclear size compared with WT Trbl (Fig. 2C,D).

**Fig. 3.**
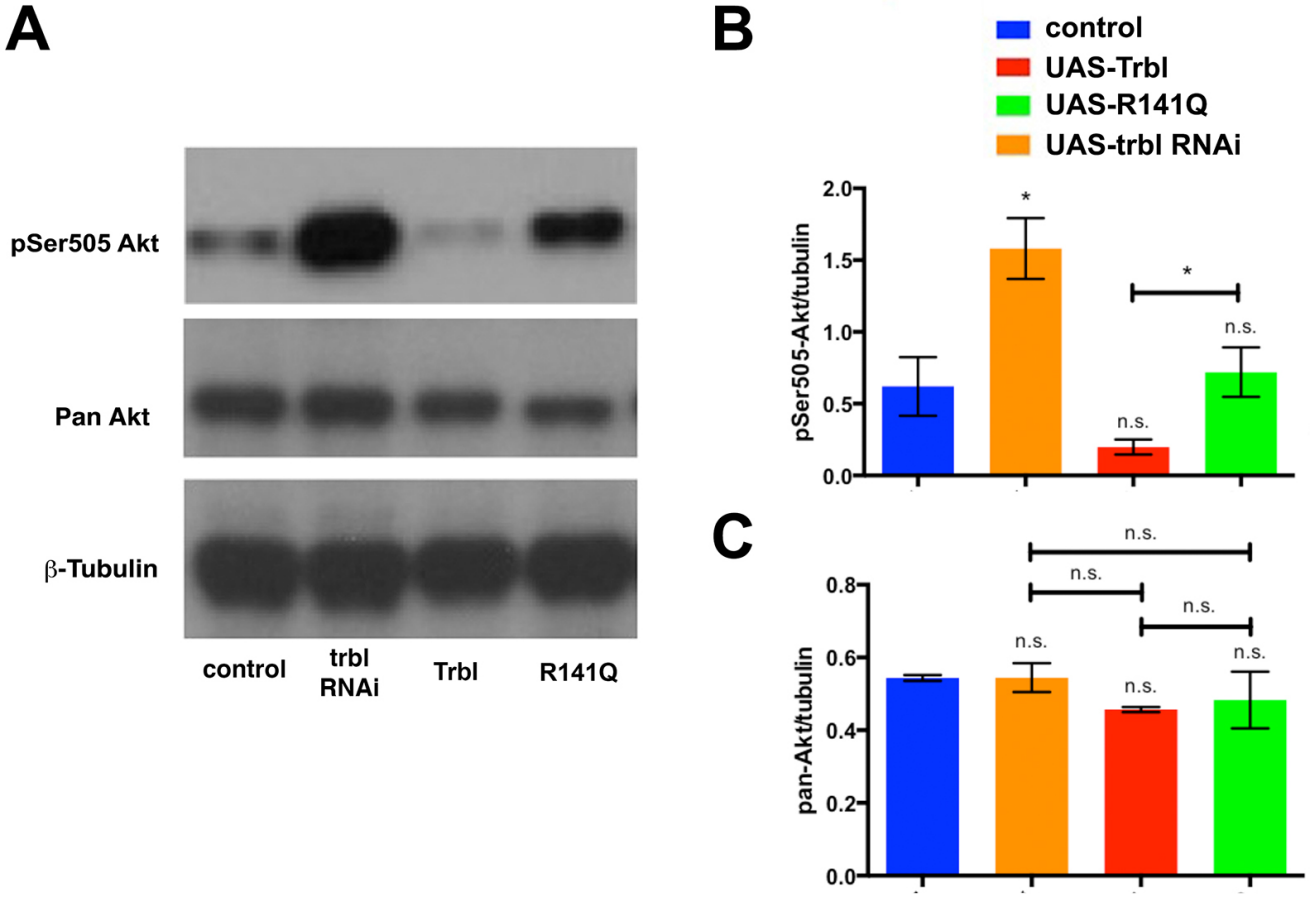
**The R141Q mutation reduced the ability of Trbl to block dAkt activation.** (A) Representative western blot of fat body extracts from age-matched larvae driving transgene expression by *r4-GAL4*, probed with phospho-Akt, panAkt and β-tubulin antisera by stripping and re-probing the same blot, showing that the R141Q mutation blocks Akt phosphorylation less effectively than WT Trbl. Genotypes: (1) control, *UAS-LacZ/+; r4-GAL4*; (2) *trib* RNAi, *P{GD11640}v22114/r4-GAL4*; (3) Trbl, *UAS-Trbl;* *r4-GAL4/+*; (4) R141Q, *UAS-trbl^R141Q^/r4-GAL4*. (B,C) Quantification of at least three independent western blots of fat body extracts shows that *trbl* RNAi increases Akt activation, whereas Trbl decreases it, and the R141Q mutation reduces the ability of Trbl to block Akt activation (B); for all three, total Akt levels were unaffected (C). β-Tubulin was used as loading control and results were normalized to control. Genotypes are the same as in A. Statistical analysis was performed with one-way ANOVA followed by Tukey post hoc using GraphPad Prism. Data are mean±s.e.m.

Compared with human TRIB3 Q84, the TRIB3 R84 variant is a stronger inhibitor of AKT-mediated insulin signaling in human hepatocytes and vein endothelial cells (Andreozzi et al., 2008; Prudente et al., 2005). To compare the effects of WT Trbl and R141Q on Akt in adipose tissue, we collected fat body tissue extracts from flies expressing these transgenes and performed western blotting with either anti-phospho *Drosophila* Ser505 Akt sera (equivalent to mammalian Ser473), to detect levels of activated Akt, or anti-pan Akt sera, to document total Akt levels. As shown in Fig. 3A, misexpression of *trbl* RNAi led to increased levels of phospho-Akt compared to controls, whereas WT Trbl misexpression effectively reduced levels of phospho-Akt. By contrast, misexpression of R141Q led to phospho-Akt levels similar to control (Fig. 3A). Quantification from multiple western blots revealed that phospho-Akt levels following WT Trbl misexpression were ∼60% lower than R141Q misexpression (Fig. 3B). As shown previously (Das et al., 2014), WT Trbl misexpression did not affect total levels of Akt, and here we show that R141Q behaved similarly (Fig. 3A,C). In summary, the R141Q mutation impaired the potent ability of Trbl to block Akt phosphorylation, consistent with its ability to block Trbl-mediated reduction of tissue growth.

### R141Q is a weaker inhibitor of insulin signaling

**Fig. 4.**
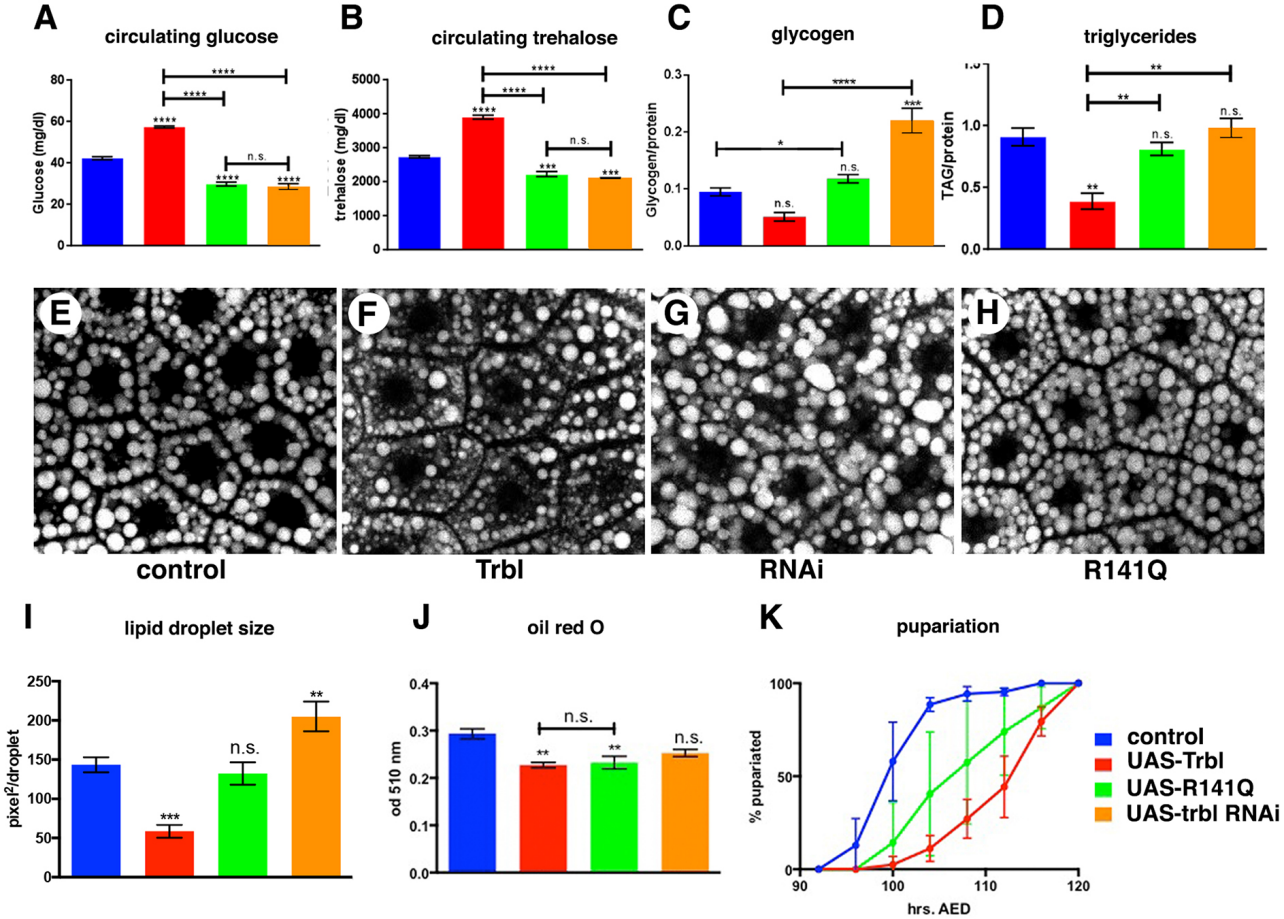
**Trbl R141Q mutation reduces Trbl inhibition of larval growth and metabolism.** (A-D) Assays for stored or circulating metabolites were performed on age-matched larvae and reveal the effect of misexpressing WT Trbl, *trbl* RNAi or R141Q in the fat body: circulating glucose (A), circulating trehalose (B), stored glycogen (C) and stored triglyceride (D). Note that triglyceride and glycogen content are presented as relative to the protein content of the tissue sample to normalize the weight difference between control and transgene-expressing larvae (*n*=20 for A-D). (E-H). Nile Red staining (detecting lipid droplets) reveals that misexpression of Trbl in the fat body decreased lipid accumulation as detected by the relative strength of fluorescence compared to the control while, conversely, *trbl* RNAi increased fluorescence. Misexpression of the R141Q mutation blunted the reduced fluorescence seen with WT Trbl. Note that all images were taken with identical confocal parameters so that the strength of staining is comparable. (I) Quantitation of lipid droplet size in fat body cells from E-H demonstrates the strong increase in lipid droplet size caused by misexpression of *trbl* RNAi and the converse reduction in droplet size by misexpression of Trbl, which is attenuated by the R141Q mutation. (J) Oil Red O binding (detecting neutral lipids, in arbitrary units) reveals that misexpression of Trbl in the fat body decreased lipid accumulation compared to the control, while *trbl* RNAi misexpression had the opposite effect and increased lipid accumulation significantly. In this assay, the effect of misexpression of the R141Q mutation was not significantly different from that of misexpression of Trbl. (K) In comparison to controls, fat body misexpression of WT Trbl delayed pupariation, whereas R141Q misexpression did not delay pupariation as effectively. Genotypes: (1) control, *r4-GAL4/+*; (2) Trbl, *UAS-trbl;* *r4-GAL4*; (3) R141Q, *UAS-trbl^R141Q^/r4-GAL4*; (4) *trib* RNAi, *P{GD11640}v22114/r4-GAL4.* All experiments were performed in biological triplicate. *n*=60, *n*=3, *n*=20 in I, J and K, respectively. For all metabolite quantitation, lipid droplet analysis, and pupariation assays, statistical analysis was performed with one-way ANOVA followed by Tukey post hoc in GraphPad Prism. Data are mean±s.e.m.

In vertebrates, hepatic Trib3 misexpression in mice leads to hyperinsulinemia, owing to its ability to block Akt-mediated glucose uptake (Du et al., 2003; Matsushima et al., 2006). To compare the effect of WT Trbl and the R141Q mutation on circulating sugar levels, we collected hemolymph from larvae misexpressing either WT Trbl or R141Q in the fat body. Misexpression of WT Trbl increased the circulating glucose levels by ∼30% compared with the control. By contrast, R141Q misexpression did not significantly increase circulating glucose concentration (Fig. 4A). Next, we examined circulating levels of trehalose, a glucose disaccharide that constitutes the major form of circulating sugar in insects (Thompson, 2003). WT Trbl misexpression increased circulating the trehalose levels by ∼40%, but R141Q did not change trehalose levels significantly (Fig. 4B). This could reflect a reduction in the ability of R141Q to block the clearance of sugars from the hemolymph in the body wall muscles, the major storage site of glycogen in *Drosophila* larvae (Ruaud et al., 2011). To rule out this possibility, we measured larval glycogen content and found that neither WT Trbl nor R141Q misexpression in the fat body altered overall larval glycogen levels significantly compared with the control (Fig. 4C). Taken together, these data indicate that the R141 residue is important for Trbl-mediated carbohydrate clearance from the hemolymph.

The R141Q mutation reduced the potent ability of Trbl to decrease fat body cell size and larval body weight (Fig. 2A-D), so we next examined its effects on adipose tissue fat deposit. As previously shown (Das et al., 2014), WT Trbl misexpression decreased the levels of fat body triglycerides, the major constituent of the stored fat in insects (Canavoso et al., 2001). By contrast, R141Q misexpression did not alter triglyceride levels significantly (Fig. 4D), a result that might explain the normal pupariation rate in these larvae. Consistent with opposing effects on fat stores, WT Trbl misexpression in the fat body resulted in reduced lipid droplet size, whereas *trbl* RNAi increased droplet size compared to control (Fig. 4E-G). R141Q misexpression resulted in droplet size that was significantly larger than Trbl, consistent with its reduced activity (Fig. 4H,I). Similarly, R141Q had stronger Nile Red fluorescence, which detects lipid droplets in fat bodies and emits fluorescence in the 552/636 nm range, compared with Trbl (Fig. S2). Consistent with these data, R141Q showed increased retention of Oil Red O stain, which detects neutral lipids, compared with Trbl misexpression (Fig. 4J).

When body weight reaches a critical threshold necessary to sustain metabolic needs prior to pupariation, third instar larvae cease feeding and wander away from the food, and insulin signaling regulates the timing of these events (Shingleton, 2005). As we showed before, WT Trbl misexpression in the fat body using the *r4-GAL4* driver delayed the timing of pupariation, consistent with its ability to block insulin signaling (Das et al., 2014). By contrast, misexpression of R141Q did not alter the pupariation rate significantly compared with control larvae (Fig. 4K), consistent with the notion that R141Q is a weaker inhibitor of insulin signaling than R141. These data indicate that the R141 motif plays a key role in nutrient and energy homeostasis during development.

### The nature of the charged residue at R141 mediates the strength of Akt binding

**Fig. 5.**
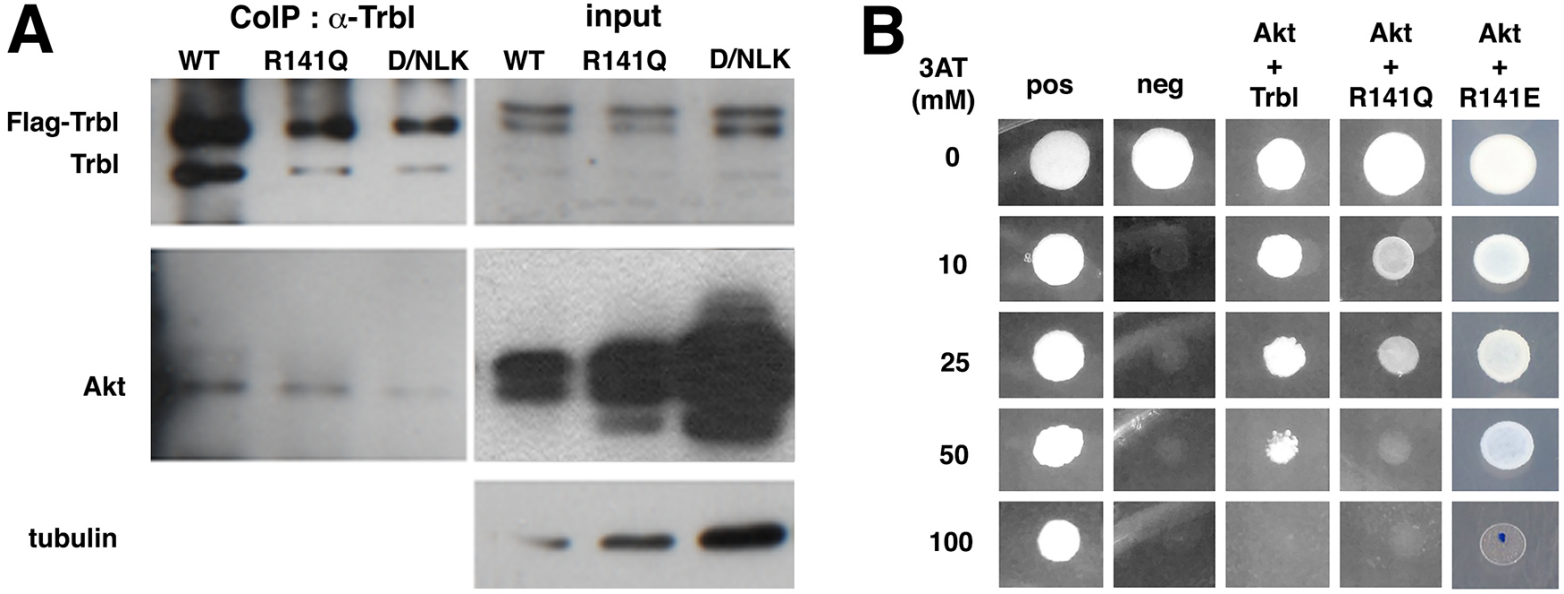
**The R141Q mutation reduces Akt binding.** (A) Western blot probed with pan-Akt antisera reveals that Akt co-immunoprecipitates with Trbl in embryonic extracts misexpressing either Trbl or R141Q, and shows reduced Akt binding to R141Q compared to WT Trbl. The DLK mutation in the kinase domain of Trbl strongly reduced Akt interaction, as shown previously (Das et al., 2014). (B) Y2H assay of Trbl prey shows strong Akt bait binding that is reduced by the R141Q mutation, while the R141E mutant bound Akt more strongly in this assay. Yeast cells co-expressing Akt prey and R141Q bait were able to grow well in the presence of ≤25 mM 3AT growth inhibitor, whereas cells co-expressing Akt and WT Trbl grew in the presence of ≤50 mM 3AT, and cells co-expressing Akt and R141E bait grew in the presence of ≤50 mM 3AT. Note that equal numbers of transformed yeast cells were used to seed the plates used for assay.

Next, we compared the strength of WT Trbl and R141Q binding to Akt by co-immunoprecipitation (co-IP) analysis *in vivo* and *in vitro*, and by yeast two-hybrid (Y2H) assay. Embryonic extracts misexpressing *Drosophila* Akt (dAkt) and either WT Trbl, R141Q or a D/NLK mutation in the catalytic loop of Trbl (Das et al., 2014) were subjected to immunoprecipitation with Trbl antisera followed by western blot analysis using anti-Akt sera. As shown in Fig. 5A, we detected considerably less Akt associated with the same amount of R141Q than with WT Trbl. The D/NLK mutation in the catalytic loop, which reduced Trbl function in several tissues (Das et al., 2014), reduces further the strength of this interaction with Akt (Fig. 5A).

To further confirm these data, we cloned WT Trbl, R141Q and dAkt coding sequences into a T7 RNA polymerase-inducible vector and used a rabbit reticulocyte extract to produce each protein using coupled transcription/translation. Fluorescently labeled lysine was incorporated during expression of dAkt and extracts were mixed with WT Trbl or R141Q immobilized on nickel beads, and bound Akt was eluted by boiling in SDS loading buffer. Following polyacrylamide gel analysis, fluorescent Akt levels were measured directly using a laser-based scanner. As shown in Fig. S3, comparably less labeled Akt was bound to R141Q than to WT Trbl following pulldown in this *in vitro* interaction assay.

To directly compare the strength of interactions between Trbl and Akt in the absence of endogenous proteasomal degradation, we cloned WT Trbl or R141Q into a Y2H bait vector and dAkt in the corresponding prey vector. As shown in Fig. 5B, WT Trbl bait interacts with dAkt prey more strongly than R141Q bait, resulting in detectable growth in the presence of ≤50 mM 3-amino-1, 2,4-triazole (3AT), whereas R141Q could grow well only in the presence of ≤25 mM 3AT (Fig. 5B). These results agree with observations in the mammalian system, in which human R84 TRIB3 binds more strongly to AKT than its Q84 counterpart (Andreozzi et al., 2008).

### Mouse Trib3 has conserved activity to block Akt activation

**Fig. 6.**
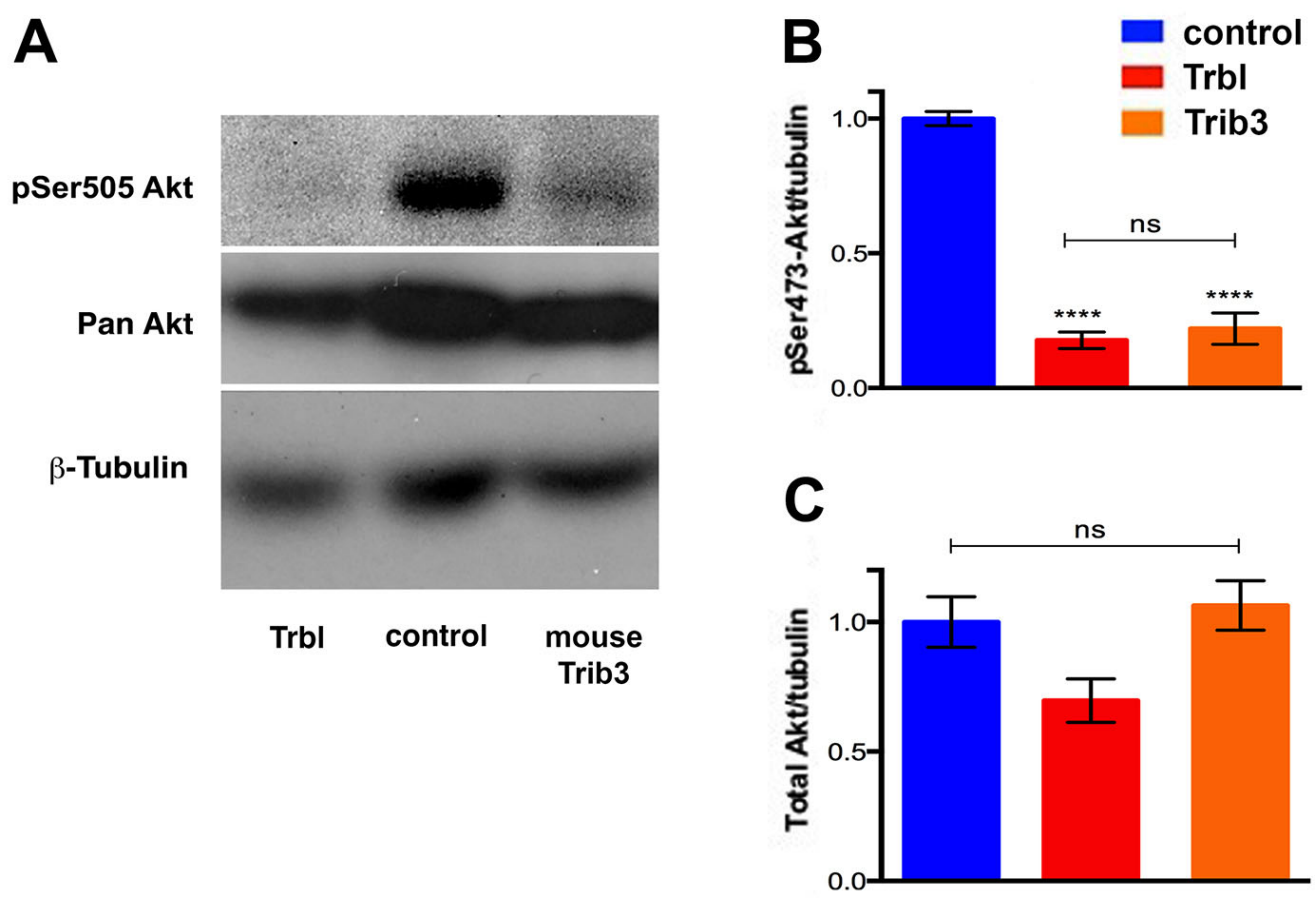
**Mouse Trib3 functions in a conserved manner to block dAkt activation.** (A) Representative western blot of fat body extracts from age-matched larvae driving expression of UAS-Trbl or UAS-Trib3 from mice by *r4-GAL4*, probed with phospho-Akt, panAkt and β-tubulin antisera by stripping and re-probing the same blot, reveals that mouse Trib3 was as effective as Trbl in blocking Akt phosphorylation. Genotypes: control, *r4-GAL4/+*; Trbl, *UAS-trbl/+; r4-GAL4/+*; Trib3, *UAS-mouseTrb3/+; r4-GAL4/+.* (B,C) Quantification of western blots of fat body extracts from at least three independent experiments shows that mouse Trib3 is as effective as Trbl in reducing Akt activation but has no strong effect on total Akt levels. α-Tubulin was used as a loading control and results were normalized to the control. Genotypes are the same as in A. Statistical analysis was performed with one-way ANOVA followed by Tukey post hoc using GraphPad Prism. Data are mean±s.e.m.

These observations confirm the importance of a conserved arginine residue for binding and blocking Akt activation, and led us to test the effect of misexpressing a mouse Trib3 bearing this conserved R84 in the *Drosophila* model (Fig. 6). As shown in Fig. 6A-C, misexpression of mouse Trib3 in the fat body resulted in a decrease in pSer505 levels, while total dAkt levels were unaffected, two effects that were similar to those observed with WT Trbl. The ability of mouse Trib3 R84 to block Akt activation in the fly model demonstrates the functional importance of this conserved arginine for Trib family member regulation of insulin-mediated metabolism.

### Mutational analysis of the R141 site

To further test the importance of the R141 site for Akt interaction, we generated a set of site-directed mutations in the R141 site and used the Y2H system to test the strength of Trbl-Akt binding (R.D., A.S., J-Y.F. and L.L.D., unpublished data). From this pilot screen, we identified a charge reversal mutation R141E in Trbl bait that interacted with dAkt in the prey vector more strongly than WT Trbl bait, resulting in strong growth in the presence of ≤50 mM 3AT compared with WT Trbl bait, which had only weak growth in 50 mM 3AT (Fig. 5B). We designed the corresponding site-directed mutation in the UAS vector and misexpressed Trbl^R141E^ (R141E) in the fat body using the *r4-GAL4* driver to test its effect on Akt binding and regulation.

Trbl^R141E^ protein accumulated in the fat body with a subcellular distribution similar to WT Trbl, as detected by Trbl antisera (Fig. S4A). Confirming the Y2H result, R141E purified from fat bodies bound Akt more strongly than WT Trbl in a pulldown co-IP assay (Fig. S5B). This increase in Akt binding compared to WT Trbl suggested that R141E might have strong dominant properties *in vivo*. However, misexpression in the fat body reduced overall larval body weight and fat body cell size less significantly than WT Trbl (Fig. S5B,C). Consistent with these weaker effects, R141E was less effective than WT Trbl at reducing levels of phospho-Akt *in vivo* (Fig. S6). The effects of R141E misexpression in the fat body on metabolism were more complex, with effects similar to WT Trbl on total triglyceride storage (Fig. S7A), but with reduced effects on glycogen (Fig. S7B), circulating glucose (Fig. S7C) and trehalose levels (Fig. S7D) compared to WT Trbl. Moreover, the effect of R141E on delaying the timing of pupariation was identical to that of Trbl (Fig. S7E). This evidence suggests that although the R141 site is important for Akt interactions, its effects on metabolism *in vivo* might reflect its interaction with other protein targets as well.

### R141Q mutation does not affect other Trbl developmental functions

In vertebrates, the Q84R mutation in Trib3 confers greater protein stability, increased MAPK activity, and increased binding to the ATF4 transcription factor (Formoso et al., 2011; Liew et al., 2010), while an R/L mutant in Trib1 enhances ERK phosphorylation and C/EBPα degradation. These data suggest that this site can mediate the interactions between Trib family members and a wide variety of targets (Yokoyama et al., 2012), and led us to test whether the R141Q or R141E mutations affected Trbl-dependent processes in other fly tissues.

**Fig. 7.**
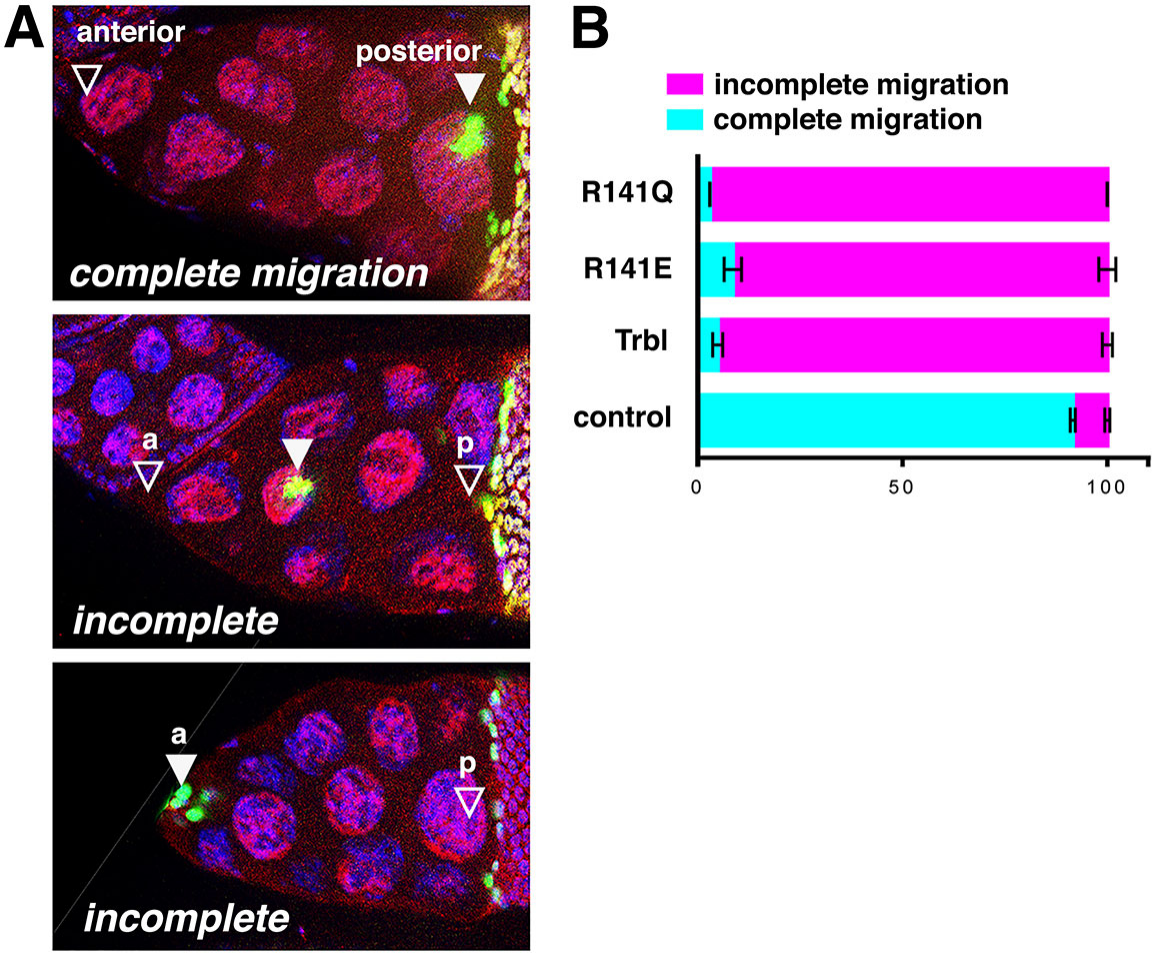
**Mutations at the R141 site do not affect** **BC** **migration.** (A) *slbo2.6-GAL4* misexpression of Trbl, R141Q or R141E at 30°C resulted in a variable block in BC migration (complete migration and two examples of incomplete migration; anterior and posterior empty arrowheads show the start and end of migration, respectively; filled arrowheads show BC migration progress). (B) Quantitation of the block to BC migration reveals a strongly penetrant incomplete BC migration phenotype for Trbl, R141Q and R141E at stage 10B. Genotypes: (1) control, *slbo2.6-GAL4, UAS-GFP/UAS-lacZ*; (2) Trbl, *slbo2.6-GAL4, UAS-GFP/UAS-trbl*; (3) R141Q, *slbo2.6-GAL4, UAS-GFP/UAS-trbl^R141Q^*; (4) R141E, *slbo2.6-GAL4, UAS-GFP/UAS-trbl^R141E^*. Statistical analysis was performed with one-way ANOVA. Data are mean±s.e.m.

The *Drosophila* C/EBP transcription factor homolog Slow border cells (Slbo) promotes border cell (BC) migration during oogenesis, and WT Trbl misexpression in these cells potently blocks their migration by directing the proteasomal degradation of Slbo (Rørth et al., 2000). Misexpression of R141Q and R141E specifically in the BCs using *slbo2.6-GAL4* driver elicited a comparable strong block on BC migration (Fig. 7A,B). In support of this *in vivo* functional assay, in a Y2H binding assay, the bait proteins WT Trbl, R141Q and R141E all interacted with prey Slbo, with an approximate order of strength of WT Trbl=R141E>R141Q (Fig. S4A).

**Fig. 8.**
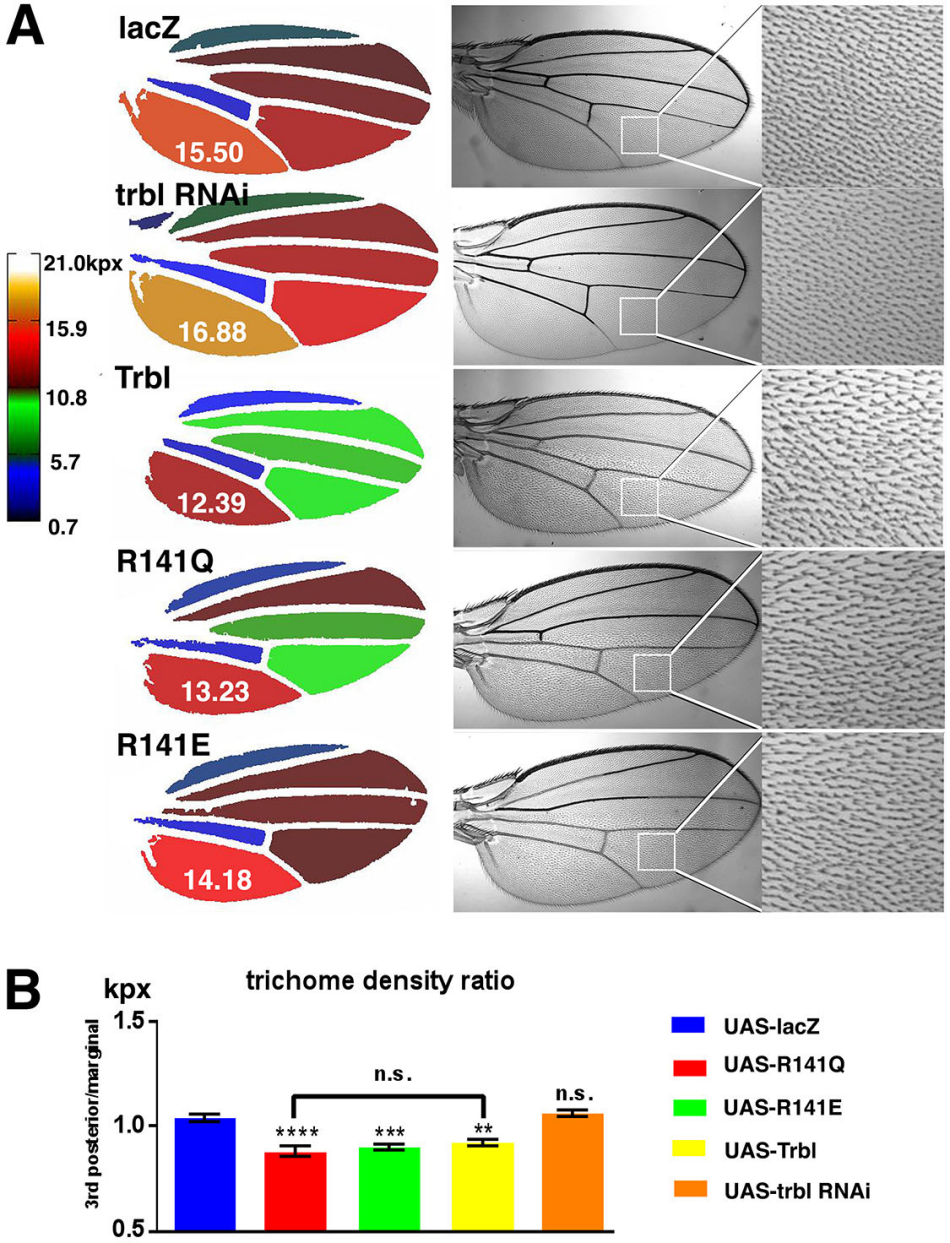
**Mutations at the R141 site do not affect Trbl-regulated tissue growth.** (A) *engrailed-GAL4* (*enGAL4*) misexpression of WT Trbl, R141Q or R141E resulted in no strong differences in overall reduced tissue growth. Overall wing size represented by heat map diagrams of typical wings and the area of the posterior intervein region which is directly affected by *enGAL4* transgene misexpression in the representative wing is indicated in kilopixels. (B) *enGAL4* misexpression of R141Q or R141E blocked cell division as effectively a misexpression of Trbl. There was no significant difference in the effect of *engrailed-GAL4* misexpression of WT Trbl, R141Q and R141E at 30°C to increase cell size as detected by decreased trichome density in the third posterior compartment (normalized to density of trichomes in the marginal compartment). Data are mean±s.e.m. (*n*=10 for control, *n*=7 for WT Trbl and R141Q). Genotypes: (1) control, *en-GAL4/UAS-lacZ*; (2) Trbl, *en-GAL4/UAS-Trbl*; (3) R141Q, *en-GAL4/UAS-trbl^R141Q^*; (4) R141E, *en-GAL4/UAS-trbl^R141E^*. Statistical analysis was performed with one-way ANOVA followed by Tukey post hoc using GraphPad Prism.

Trbl inhibits cell division by binding and promoting degradation of String/Cdc25 phosphatase and misexpression of Trbl in the wing blocks cell division so that the density of trichomes, actin-rich protrusions projecting out from the distal edge of each wing cell, is noticeably reduced. At the same time, Trbl block of Akt activity reduces the overall size of the wing (Dobens and Dobens, 2013; Grosshans and Wieschaus, 2000; Mata et al., 2000). Previously, we used the Fijiwings program to demonstrate that WT Trbl misexpression in the wing results in increased cell size and decreased overall tissue growth (Dobens and Dobens, 2013). When we used the *engrailed-GAL4* driver to compare the effects of WT Trbl, R141Q or R141E misexpression in the wing (Fig. 8A,B), we observed that R141Q or R141E resulted in a reduction in overall wing size and trichome density ratio, similar to the effect of WT Trbl. These observations in two tissues suggest that the R141 residue, which plays a crucial role in binding and preventing Akt activation, does not affect the interaction of Trbl with its targets Slbo (C/EBP) and String/Cdc25.

## DISCUSSION

We reported previously that fly Trbl has a conserved role in modulating Akt to regulate the insulin signaling pathway (Das et al., 2014) and, here, we extend these data by demonstrating that a conserved site in Trbl corresponding to an SNP variant R84 in human TRIB3, which is associated with insulin resistance, is important in the modulation of insulin signaling in flies as well (Prudente et al., 2005). Functional studies in human cell and organ culture demonstrate that the TRIB3 variant Q84 is a weaker binder and inhibitor of AKT than the R84 variant (Andreozzi et al., 2008; Prudente et al., 2005), and here we report that when the equivalent residue R141 of *Drosophila* Trbl is mutated to Q (R141Q) a parallel effect is seen: reduced Trbl binding to Akt (Fig. 3), with consequent increases in larval growth and anabolism (Figs 2, 4 and 5). Collectively, these observations suggest that the R residue has a conserved role in binding Akt to block its activation and, consistent with this notion, we found that mouse Trib3 (an R variant Trib) misexpressed in *Drosophila* fat body inhibits Akt activation to an equivalent extent as WT Trbl (Fig. 6).

The Q residue prevalent in human populations is unique to humans and Neanderthal sequences, but the R residue is conserved in almost all metazoan Trib proteins, indicating that the TRIB3 Q84 variant appeared only late during human evolution. Notably, TRIB1 and TRIB2 orthologs in humans retain the R residue, pointing to its important role in family member function. Although the detrimental effect of the Q/R variant has been documented (Prudente and Trischitta, 2015), an intriguing question is the selective role this motif played in the evolution of human and Neanderthals. Because short-term nutrient deprivation causes an increase in TRIB3 mRNA and protein levels in adipose tissue (Liu et al., 2012), and in human cell culture TRIB3 expression level is induced upon starvation of glucose and amino acids (Schwarzer et al., 2006), one possibility is that the Q84 variant permits residual Akt activity during prolonged physical activity, such as endurance running, a capability that distinguishes the hominid lineage (Bramble and Lieberman, 2004).

In addition to the SNP that results in the Q/R variant in human populations, a number of nonsynonymous SNPs in the genomic region encompassing the human TRIB3 gene occur, the function and potential biological relevance of which are, up to now, unaddressed. The fly insulin resistance model offers a convenient place to test the stage-, tissue- and diet-specific effects of these Trib3 SNPs (Das and Dobens, 2015). Because Trib3 is regulated by diet, testing Trbl function on various high sugar and high fat diets can be performed rapidly (Musselman et al., 2011). Vertebrate Trib3 has tissue-specific effects on liver, endothelium and pancreatic islets, and because these tissues have parallel organ systems in the fly, the power of the genetic tools available in the fly to misexpress or knockdown Trbl can be implemented. Furthermore, the documented role of TRIB3 as a nexus between diabetes and cancer (Izrailit et al., 2013; Mondal et al., 2016) can inform tests of Trbl in recently developed fly insulin-regulated tumor models (Figueroa-Clarevega and Bilder, 2015; Kwon et al., 2015).

Mutations in the conserved DLK motif strongly reduce the interaction of Tribbles family members with Akt, C/EBP and Cdc25 phosphatase (String) in flies and vertebrates (Das et al., 2014; Du et al., 2003; Keeshan et al., 2010; Liang et al., 2016; Masoner et al., 2013). Here, we show that the R141Q mutation does not interfere with the ability of Trbl to inhibit Cdc25 phosphatase-triggered cell division or C/EBP-mediated cell migration (Fig. 8), suggesting that this motif might be a good potential therapeutic target to selectively alleviate insulin resistance without affecting the other cellular functions of Trbl. The contribution of Tribbles isoforms in mammals to insulin regulation and cancer is complex: recently, TRIB2 has been shown to be an Akt agonist (Hill et al., 2017), and an R107L mutation in TRIB1 that aligns with R141Q in TRIB3 promotes leukemogenesis by enhancing ERK phosphorylation and C/EBPα degradation (Yokoyama et al., 2012).

Represented by a single Tribbles isoform, the fly model offers a promising model system to rapidly interrogate the molecular defects associated with human Tribbles disease alleles to accelerate an understanding of their contributions to Type 2 diabetes and cancer (Alfa and Kim, 2016). Toward this end, we used *in vitro* Y2H interaction assays to identify an R141E charge reversal mutation that binds Akt more strongly, and then engineered this mutation into an inducible transgene to test it in the fly. We confirmed the strong interaction between R141E and Akt *in vivo*, suggesting that the allele might have dominant activity, effectively blocking Akt activity more potently than WT Trbl. However, the allele actually reduced Trbl activity measured by cell and tissue size, Akt phosphorylation and metabolic assays. It is possible that the R141E mutation affects interactions with other Trbl targets regulating metabolism, such as acetyl-coenzyme A carboxylase (ACC), which our preliminary data show is a conserved Trbl target regulating fat production (Liew et al., 2010; Qi et al., 2006). Thus, while this R141E mutation confirms the crucial role of this site in Akt binding, its overall influence on metabolism reflects the complexity of even the simpler fly system.

## MATERIALS AND METHODS

### *Drosophila* strains

Generation of (1) *P{UAS-FLAG-trbl.WT}* (*UAS-FLAG-trbl*) was described previously (Masoner et al., 2013). Generation of (2) *P{UAS-FLAG-trbl^R141Q^}* (141Q), (3) *P{UAS-FLAG-trbl^R141E^}* (141E), and (4) *P{UAS-FLAG- mouseTrib3}* are described below. (5) y^1^ w*; P{r4-GAL4}3 was a generous gift from Laura Musselman (Binghamton University, Binghamton, NY). We obtained the following stocks from Bloomington *Drosophila* Stock Center: (6) *P{en2.4-GAL4}e16E* (*engrailed-GAL4*); (7) *P{w*(+mC)*=UAS-lacZ.NZ}20b* (Rulifson et al., 2002); (8) *y*(1) *w; P{w*(+mC)*=UAS-Akt1.Exel}2*. We obtained (9) *w1118; P{GD11640}v22114* from Vienna *Drosophila* RNAi Center. (10) *{GAL4-slbo.2.6}1206 P{UAS-GFP.S65T}T2/CyO* (*slbo2.6-GAL4*) was a generous gift from Pernille Rorth (Institute of Molecular Cell Biology, Singapore).

### Construction of *P{UAS-FLAG-mouseTrib3}*

The complete ORF mouse *trib3* was amplified from cDNA (Origene) using the oligonucleotides 5′-ATG**GATTACAAGGATGACGACGATAAG**ATGCGAGCTACACCTCTG-3′ and 5′-TCAGCCGTACAGCCCCACCTCCCCTTC-3′ to generate an N-terminal FLAG fusion (FLAG sequence in bold), cloned into pSTBlue-1 (AccepTor Vector kit, Novagen) and confirmed by DNA sequencing. An EcoRI fragment containing the full-length FLAGTrbl and FLAGTrib3 was then cloned into pUASTattB and again confirmed by DNA sequencing.

### Construction of *UAS-FLAG-trbl^R141Q^* and *UAS-FLAG-trbl^R141E^*

Mutated versions of Trbl were generated from pUASTattB+FLAG-*trbl.*WT plasmid using the QuikChange II XL Site-Directed Mutagenesis Kit (Stratagene). Oligonucleotides designed were as follows: for *UAS-FLAG-trbl^R141Q^*, 5′-CTAACCGCCTCCAATCTGC**AG**TGCGTGGACATCTTCACC-3′ and 5′-GGTGAAGATGTCCACGCA**CT**GCAGATTGGAGGCGGTTAG-3′; for *UAS-FLAG-trbl^R141E^*, 5′-CTAACCGCCTCCAATCTG**GAA**TGCGTGGACATCTTCACC-3′ and 5′-GGTGAAGATGTCCACGCA**TTC**CAGATTGGAGGCGGTTAG-3′. Site-directed mutations (shown in bold) were confirmed by DNA sequencing. Embryo injections were performed as a fee-for-service (Genetic Services, Cambridge, MA and Rainbow Transgenic Flies, Thousand Oaks, CA), and transgenic lines with insertions recombined into the second chromosome landing site (attP40; 25C7 on 2L) or third chromosome landing site (attP2; 68A4 on 3L) were confirmed for the presence of the WT or mutant transgene by sequencing of PCR product.

### Y2H screen

The ProQuest Two-Hybrid System (Invitrogen) was used to perform Y2H interaction tests. Plasmid DNA transformations of *Saccharomyces cerevisiae* strain MaV203 were performed as outlined in the kit manual and grown on synthetic complete (SC) media lacking both tryptophan and leucine to confirm the presence of the bait and prey plasmids. An equal number of transformed cells (determined by OD_600_) were transferred to SC-Leu-Trp-His, SC-Leu-Trp-His+3AT (at various concentrations) plates to test for interactions. Plate images were taken after 3-4 days of growth at 30°C. To construct bait and prey plasmids, cDNAs for FLAGTrbl WT, FLAGTrbl^R141Q^, dAkt and Slbo ORFs were amplified using PCR to add flanking attB1 and attB2 sites to each, respectively. Forward and reverse primers for FLAGTrbl WT and FLAGTrbl^R141Q^ were 5′-**GGGGACAAGTTTGTACAAAAAAGCAGGCTTC**ATGGATTACAAGGATGACGACGATAAG-3′ and 5′-**GGGGACCACTTTGTACAAAGAAAGCTGGGTC**TCAGCCCATGTCCACATCCGTATC-3′, respectively; for Slbo, 5′-**GGGGACAAGTTTGTACAAAAAAGCAGGCTTC**ATGCTGAACATGGAGTCGCCGCAG-3′ and 5′-**GGGGACCACTTTGTACAAGAAAGCTGGGTC**CTACAGCGAGTGTTCGTTGGTGTTG-3′, respectively; and for Akt, 5′-**GGGGACAAGTTTGTACAAAAAAGCAGGCTTCA**TGTCAATAAACACAACTTTCGACCTCAGCTC-3′ and 5′-GGGG**ACCACTTTGTACAAGAAAGCTGGGTC**CTATTGCATCGATGCGAGACTTGTG-3′, respectively (attB1 and attB2 sequences in bold). The PCR product was cloned into the donor vector pDONR-221 and then into either pDEST32 (bait vector) or pDEST22 (prey vector). All constructs were confirmed by DNA sequencing.

### Protein sequence alignment

The amino acid sequence of Trbl was obtained from flybase.org (FBgn0028978). All other protein sequences were obtained from FlyBase. The Neanderthal Trib sequences were obtained from ‘The Neanderthal genome project’ (neandertal.ensemblgenomes.org). Sequence alignment was performed with ‘Clustal Omega’ online alignment software (Sievers et al., 2011).

### Fly husbandry

Fly stocks were maintained at 25°C on standard cornmeal, molasses and yeast medium (CMYM) recommended by Bloomington *Drosophila* Stock Center. All crosses were performed at 30°C and progeny animals were reared at 30°C.

### Larval developmental age synchronization

All experiments involving larvae were performed with age-matched female larvae. Approximately 150 virgin female driver flies (aged 2-5 days) were crossed to ∼100 UAS-transgene-carrying male flies, and were reared in collection cages on CMYM supplemented with yeast paste for 2 days, with fresh food replaced every day. On day 3, flies were transferred to a fresh food plate for 30 min to get rid of any held eggs, and eggs were collected on a fresh plate for 2-3 h at 30°C. After 18 h at 30°C, first instar larvae were collected and transferred to a fresh food plate and allowed to grow until 72 h after egg deposition (AED) at 30°C, which corresponds to mid-third instar stage in WT larvae. All experiments described in this report were performed with mid-third instar female larvae 72 h AED. Female larvae can be distinguished from male larvae by the considerably smaller size of female gonads. Control larvae for each experiment were obtained by crossing the female driver line with *UAS-lacZ* males.

### Preparation of larvae for experiments

Mid-third instar larvae were washed, dried and weighed on a precision Kahn microbalance (model 4400). For the puparation assay, 30 age-synchronized larvae from each cross were collected and transferred to a new vial, and pupal formation was documented every 4 hours. This experiment was performed in biological triplicate. For statistical analyses, one-way ANOVA was performed followed by Tukey post hoc using GraphPad Prism.

### Quantification of metabolites

Triglyceride quantification was performed as previously described (Das et al., 2014). Triglyceride levels were normalized to protein levels in the sample. Glycogen quantification was performed as described previously (Musselman et al., 2011). Glycogen content was normalized to the protein content of the same samples. For protein quantification, larvae were homogenized on ice in 20 µl PBST (0.1% Tween 20)/larva with a hand-held homogenizer. Homogenates were then centrifuged at 4°C and 10,000 ***g*** for 5 min, and protein concentration was determined using a Pierce BCA protein assay kit according to the manufacturer's protocol. For hemolymph glucose and trehalose estimation, 15 female larvae were placed on a piece of parafilm, pierced with a fine tungsten needle near the mouthpart and ∼4 µl hemolymph was collected and immediately stored on ice. Then, 2 µl hemolymph was diluted 10-fold in ice-cold PBS and 5 µl of this diluted sample was mixed with 100 µl pre-chilled Thermo Inﬁnity™ glucose reagent for glucose assay. The samples were incubated at 37°C for 5 min and the OD was measured at 340 nm. For the trehalose assay, porcine kidney trehalase (Sigma-Aldrich, T8778) was added at a concentration of 1 µl/ml glucose reagent and the samples were incubated at 37°C overnight. The OD was then measured at 340 nm and free glucose concentration measurements were subtracted to determine trehalose concentration. Glucose and trehalose standards ranging from 0.08 mg/ml to 2 mg/ml, and from 0.08 mg/ml to 5 mg/ml, respectively, were used to generate a standard curve. Nile Red staining and Oil Red O assay were performed as previously described (Das et al., 2014). These experiments were performed in biological and technical triplicate. Statistical analysis was performed by one-way ANOVA followed by Tukey post hoc using GraphPad Prism.

### Western blotting and co-IP

The fat body was dissected from 30 age-matched female larvae in PBS on ice and transferred to homogenization buffer containing protease and phosphatase inhibitors (10 µl/larva). After homogenization on ice, 20 µl was removed for protein assay. The remainder of the sample was mixed with an equivalent volume of 2× sample buffer and heated at 100°C for 5 min. The samples were centrifuged at 10,000 ***g*** for 5 min and loaded onto 10% SDS-PAGE gels. SDS-PAGE resolved bands were then transferred to Amersham Hybond PVDF membrane and western blotting was performed according to a standard protocol (Cell Signaling Technology). Blots were probed with antibodies (all at 1:2000 dilution) in order as follows: (1) anti-phospho-*Drosophila* Akt (Ser505) (Cell Signaling #4054), (2) anti-Akt (pan) (C67E7) rabbit mAb (Cell Signaling #4691), (3) anti-β-tubulin (#12G10 Developmental Studies Hybridoma Bank). Blots were stripped and re-probed using the Abcam mild stripping protocol. Secondary antibodies conjugated to HRP (GeneScript) were used, and the signals were detected on Storm 840 Molecular Imager (Molecular Dynamics) using Pierce ECL Plus Western Blotting Substrate. Band quantification was performed using CLIQs version 1.0 (Total Lab), and statistical analysis was performed by one-way ANOVA followed by Tukey post hoc using GraphPad Prism. Experiments were performed in biological triplicate. For co-IP, antibody-conjugated PrecipHen (goat anti-chicken-conjugated sepharose beads, Aves Labs) was prepared by washing PrecipHen three times with co-IP buffer, and then anti-Trbl antibody (in 1:20 ratio) was added to the beads and incubated overnight with agitation at 4°C. Unbound antibodies were removed by washing three times with co-IP buffer. Larval fat body was collected from ∼100 larvae in ice-cold co-IP buffer. Samples were homogenized and the final volume was brought to 500 µl by adding fresh co-IP buffer. Samples were centrifuged at 10,000 ***g*** for 10 min to remove cellular debris and equivalent amounts of protein were used for co-IP. Tissue samples were mixed with 50 µl of PrecipHen and incubated for 30 min with agitation. The beads were removed by centrifugation at 2000 ***g*** for 3 min and the sample was incubated with 100 µl anti-Trbl antibody bound to PrecipHen for 3 h with mild agitation. After the incubation period, samples were washed three times with co-IP buffer and antibody-bound protein complexes were eluted using glycine elution buffer. For embryonic extract, embryo cages containing >50 females and males were set up with a CMYM plate with yeast paste at the bottom. Plates were removed from cages after incubating for 24 h at 25°C. Samples were collected from the plate using distilled water and a paintbrush to loosen the embryos from the media. Embryos were then collected on a fine mesh and dechorionated in a 50% bleach solution for 1 min, then washed with distilled water to remove any residual bleach. Sonicated lysate was incubated with gamma bound bead (GE Healthcare Systems) for 1 h, and spun at 10,000 ***g*** for 1 min. The suspension was transferred to a new tube, and anti-Trbl antibody was added to a 1:300 dilution, and incubated for another 2 h. The elute was mixed with 4× SDS-PAGE sample buffer and subjected to SDS-PAGE western blotting, using PVDF membrane, which was more effective for the chicken antibody. Chicken anti-Trbl antibody was used at 1:1000 dilution and BlockHen (in 1:10 dilution in PBS) was used as a blocking agent followed by HRP-conjugated secondary antibody (Pierce Clean-Blot™ IP Detection Kit-HRP).

### Fat body immunostaining

Larvae (15-20) were partially dissected in PBS to open up the body cavity and expose the fat body, and fixed in 4% paraformaldehyde in PBS for 20 min. Fixed tissue was washed three times in PBST (PBS+0.1% Triton X-100) and incubated with blocking reagent (PBST+2% normal goat serum+10% BlockHen) for 2 h at room temperature with agitation. The samples were washed three times in PBST to remove excess blocking agent and incubated with primary antibody (1:1000 chicken anti-Trbl, 1:500 rabbit anti-Akt in PBST+2.5% BSA) overnight at 4°C with agitation. Tissues were then washed three times in PBST. For the first wash, PBST was supplemented by 1:500 TRITC-conjugated phalloidin and incubated for 15 min. After three washes, tissues were incubated with secondary antibody (Alexa Fluor 488-conjugated IgG) in PBST+2.5% BSA for 2 h at room temperature with agitation. Tissues were washed twice more in PBST and incubated for 15 min in 1:1000 DAPI (Roche Diagnostics) in PBS. Finally, fat body from the ventral mid-section of each larva was collected and mounted on 75% glycerol to visualize the fluorescent staining. Micrographs were collected using an Olympus confocal laser-scanning microscope and figures prepared using ImageJ (https://imagej.nih.gov/ij/). Quantification of fat body cell size and nuclear size was performed using ImageJ and images of five larval fat bodies per group were used to measure cell and nuclear size. Statistical analysis was performed using one-way ANOVA and Tukey post hoc using GraphPad Prism.

### Wing analysis

For wing analysis, crosses were reared at 30°C and progeny female wings were selected and mounted (one wing per female was used). For Fijiwings analysis, we used FijiwingsEZ (https://sourceforge.net/projects/fijiwings/) and calculated wing size in kilopixels and trichome density in ‘per kilopixel^2^’ units (Dobens and Dobens, 2013). Statistical analysis was performed using one-way ANOVA and Tukey post hoc using GraphPad Prism.

### BC migration assay

Crosses were reared at 25°C and progeny females were fed on molasses/cornmeal media supplemented with dry yeast for 2 days to boost egg production. On day 3, ovaries were dissected from ∼20 females and stained for the GFP reporter gene expression reporter that marks BCs. The migration status of BCs from stage 10 egg chambers was determined by randomly selecting ∼50 egg chambers and observing the position of BCs. BCs at the nurse cell-oocyte boundary were considered to have completed migration, and BCs not located at the nurse cell-oocyte boundary were considered as having incomplete migration. Experiments were performed in biological triplicate. Statistical analysis was performed using one-way ANOVA and Tukey post hoc using GraphPad Prism.

### Statistical analysis

Statistical analysis was performed using GraphPad Prism (v 6.0) and one-way ANOVA followed by either unpaired, two-tailed Student's *t*-test (for analysis of two to three groups) or Tukey's post hoc analysis of significance (for more than three groups). All data are presented as mean±s.e.m. *P*<0.05 was considered significant.

### *In vitro* binding assay

pET expression vector expressing Akt (2 µg plasmid DNA) was added to T7 TNT rabbit reticulocyte mix containing fluorescently labeled lysine (Promega, WI) and incubated at 30°C for 1.5 h; *in vitro* labeled proteins were later used as an interaction probe. pET expression vector expressing Myc-tagged WT and Myc-tagged R141Q Trbl in T7 TNT rabbit reticulocyte extracts were subject to Ni-bound purification and quantitated by SDS-PAGE. An appropriate amount of Ni-bound proteins were mixed with TNT Akt probes and incubated at room temperature for 2 h. Subsequently, nonspecific binding was removed by three washes with binding buffer, and bound beads were resuspended in 1× SDS buffer and separated on SDS PAGE. Labeled Akt protein was detected on a Typhoon laser scanner (GE Healthcare Systems) after staining with Coommassie Blue or western blotting.

## Supplementary Material

Supplementary information
