# Supplementary material for: A Drosophila model of insulin resistance associated with the human TRIB3 Q/R polymorphism
Source: Dis Model Mech. 2017 Dec 1;10(12):1453–64. doi: 10.1242/dmm.030619 (PMC5769606; doi:10.1242/dmm.030619)
Supplement: Supplementary information [file dmm-10-030619-s1.pdf]

Disease Models & Mechanisms 10: doi:10.1242/dmm.030619: Supplementary information

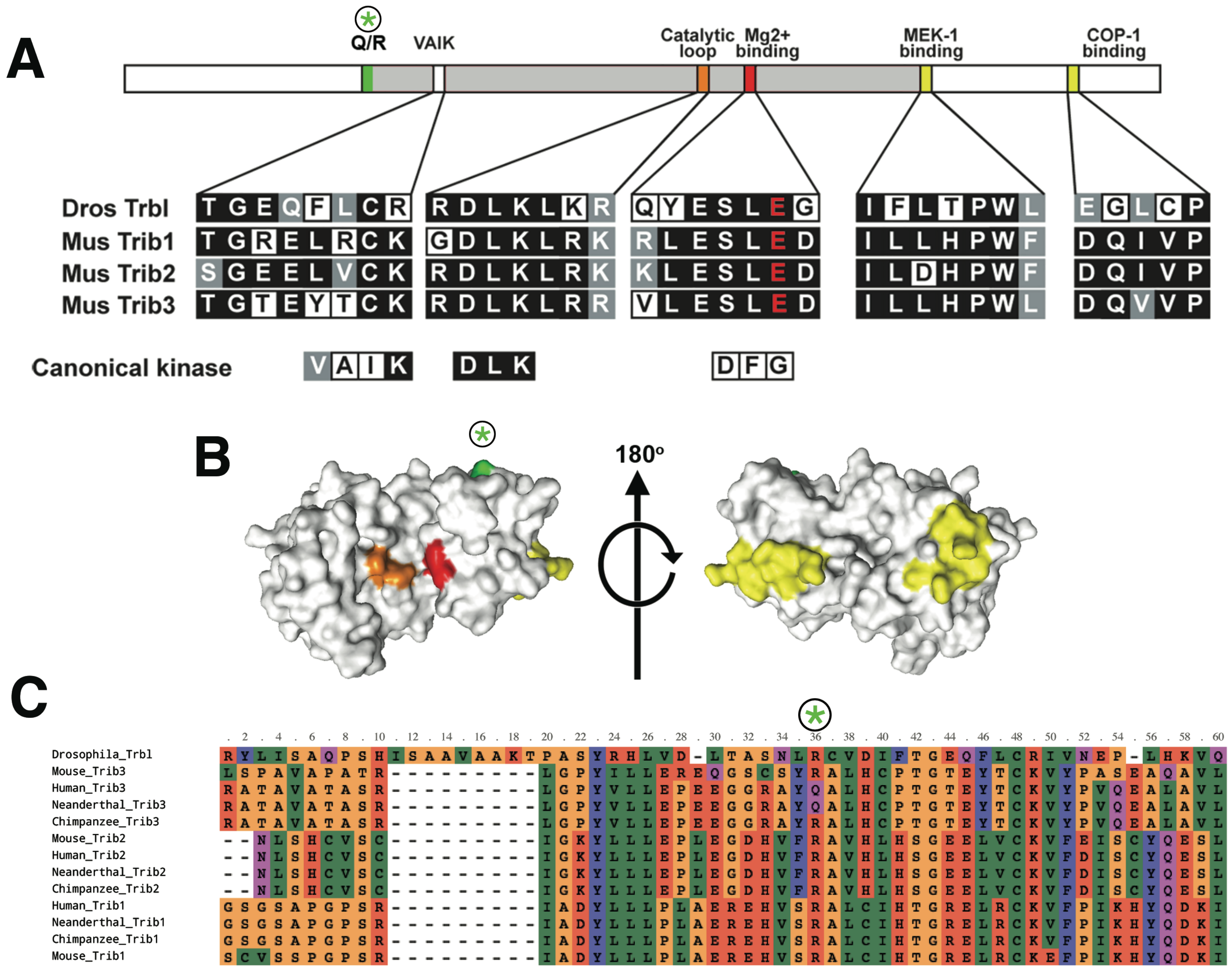

Disease Models & Mechanisms • Supplementary information

Figure S1. Human Trib3 Q84 position is highly conserved and contains R in most Trib proteins.

(A) An alignment of conserved features of the kinase domain in Tribbles family members shows where the Q/R variant motif (green) occurs.

(B) The crystal structure of human Trib1 (Murphy et al., 2015) reveals the relative locations of the conserved pocket domain (red/orange) and Q/R variant site (green). Notably, the Q/R site occurs on the surface of Trib1 three-dimensional structure.

(C) Sequence alignment of the N-terminal kinase domains of *Drosophila* Trbl with mouse, chimpanzee, Neanderthal and human Trib proteins (Alva et al., 2016) reveals the aligned positions linking the human Trib3 Q84 site to the corresponding R141 site in Drosophila Trbl, mutated here.

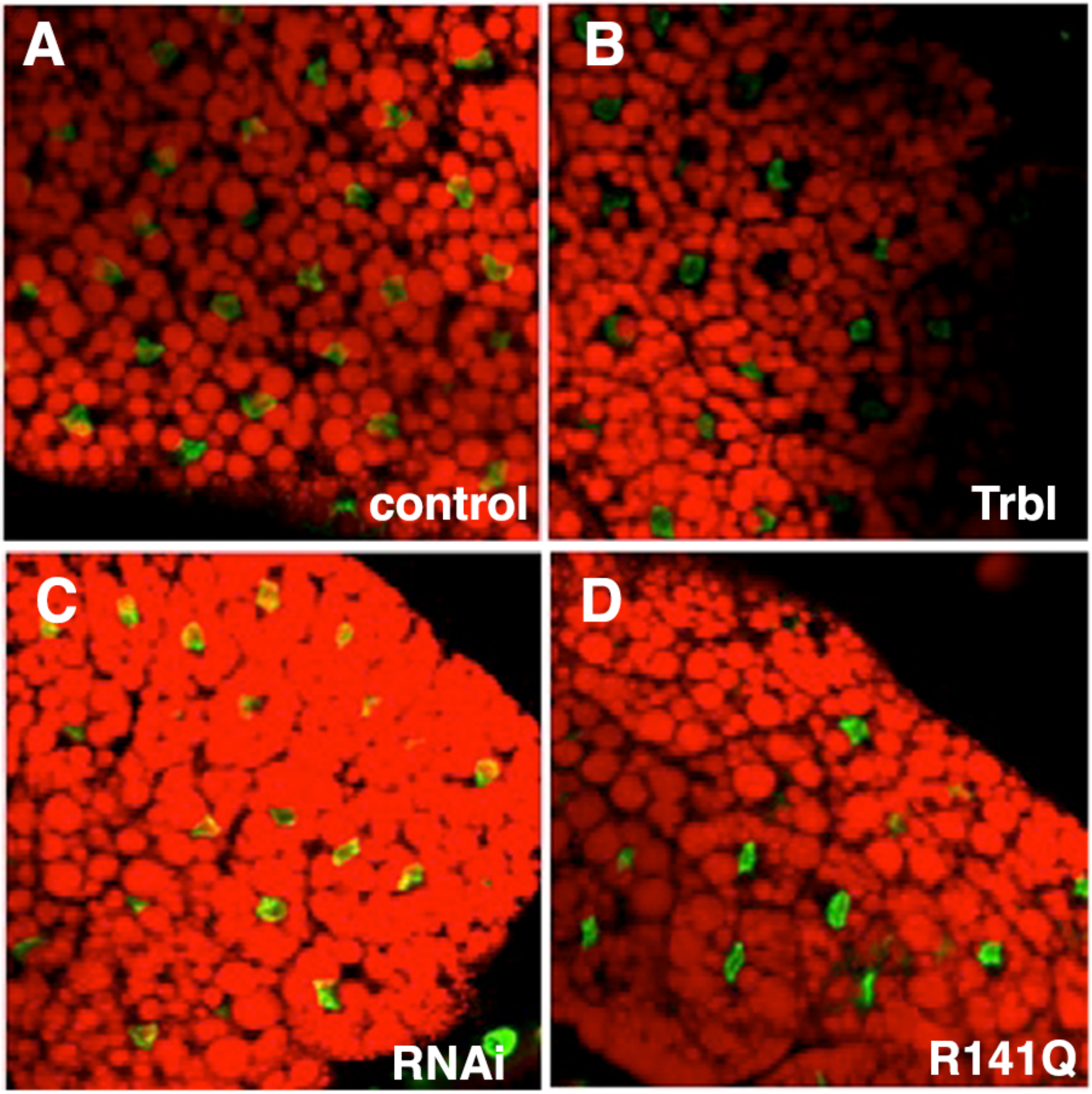

Disease Models & Mechanisms • Supplementary information

Figure S2. Trbl-mediated reduction of lipid accumulation in the fat body is attenuated by the R141Q mutation

(A-D). Nile Red fluorescent staining (detecting lipid droplets) is reduced following misexpression of Trbl in the fat body (B) compared to control (A) while conversely, trbl RNAi misexpression led to increased fluorescence (C). Misexpression of the R141Q mutation (D) blunted the effect on reduced fluorescence seen with WT Trbl (cf. B, D). All images were taken with identical confocal parameters so that the strength of fluorescent staining is comparable.

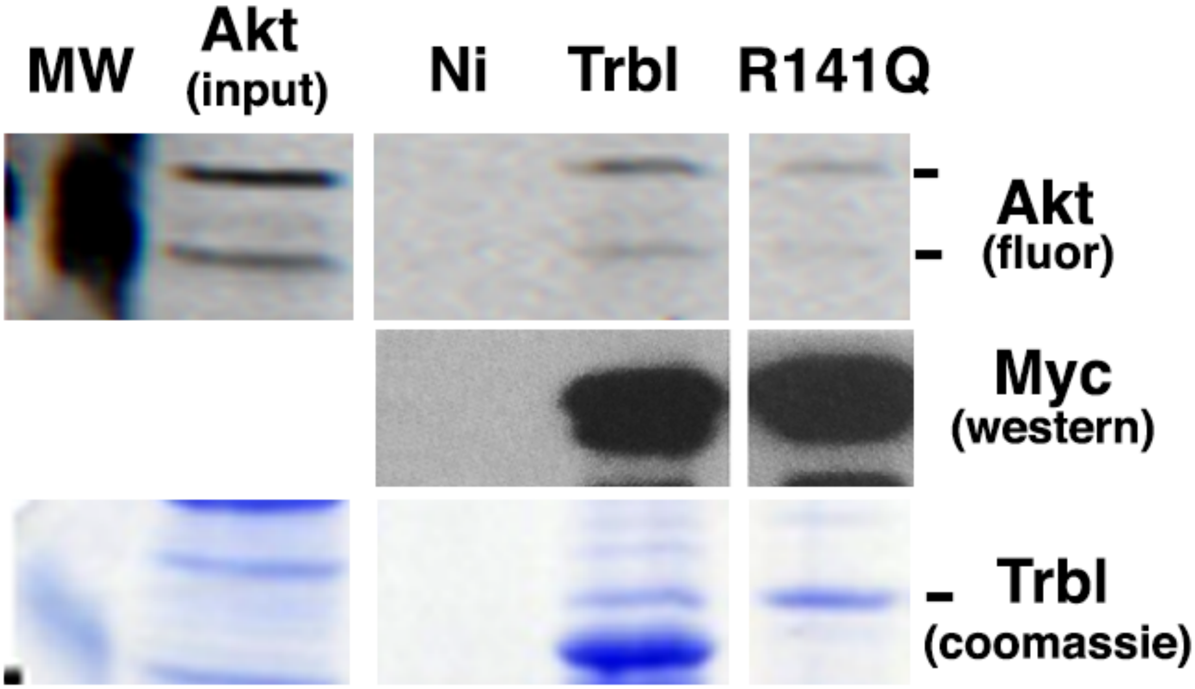

Figure S3. R141Q expressed in rabbit reticulocyte lysates shows decreased Akt binding following co-IP

(top panel) Autoradiograph exposure of gel on left showing two major labeled bands corresponding to isoform sizes of Akt (indicated). Reduced levels of labeled Akt were observed following Trbl antisera-IP of R141Q compared to Trbl.

(center panel) Western blot of gel probed with anti-MYC sera detects similar levels of Trbl and R141Q used in these assays.

(bottom panel) Coomassie brilliant blue (coomassie) staining showing total input protein from rabbit reticulocyte mixes of Trbl and labeled Akt; input band corresponding to Trbl and R141Q is indicated. These experiments were done in triplicate.

Disease Models & Mechanisms • Supplementary information

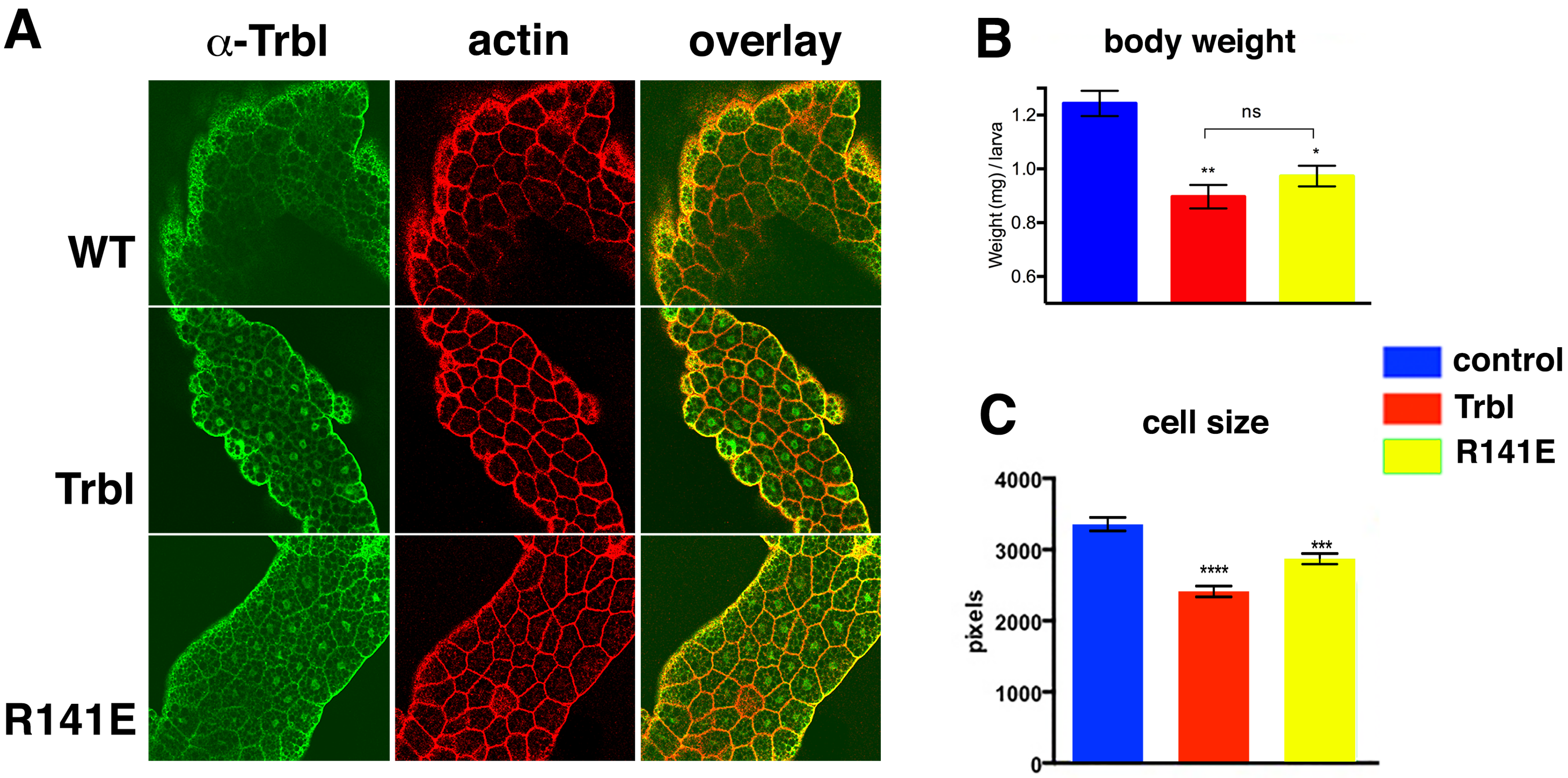

Disease Models & Mechanisms • Supplementary information

Figure S4. The R141E mutation compromises Trbl-mediated reduction in body size and fat body cell size

(A) Optical cross section comparing fat body misepression of *UAS-lacZ* (control), Trbl Trbl or R141E and immunostained for Trbl protein levels and distribution using specific antisera; actin counterstaining highlights cell size. Trbl and the R141E mutation have similar levels and distribution. Genotype: (1) control, *r4-GAL4*/+, (2) Trbl, *UAS-Trbl*/+*; r4-GAL4*/+, (4) R141E, *UAS-Trbl*^{R141E}/+*; r4-GAL4*/+. Note that all images were taken with identical confocal parameters so that the strength of staining is comparable.

(B) Age-matched larval body weight analysis shows no significant difference between the effect of Trbl and R141E on reduced body weight (n.s.).

(C) Misexpression of R141E in fat body of age-matched larvae reduced fat body cell size less significantly than WT Trbl. Statistical analysis was performed with one-way ANOVA followed by Tukey post-hoc on Graph Pad Prism. Error bars represent mean ± SEM. (B), n=20; (C), n=60.

Disease Models & Mechanisms • Supplementary information

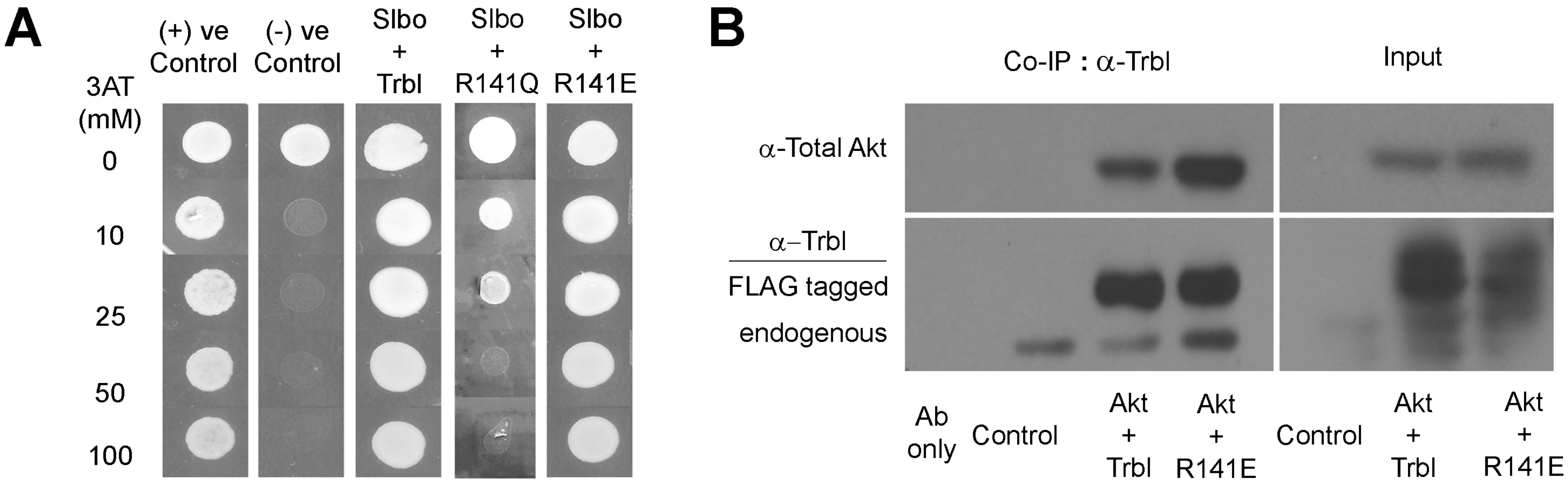
A
(+) ve Control
(-) ve Control
Slbo + Trbl
Slbo + R141Q
Slbo + R141E
3AT (mM)
0
10
25
50
100
B
Co-IP : α-Trbl
Input
α-Total Akt
α–Trbl
FLAG tagged
endogenous
Ab only
Control
Akt + Trbl
Akt + R141E
Control
Akt + Trbl
Akt + R141E

Figure S5. R141E interacts with Slbo (C/EBP) and Akt with a stronger affinity than R141Q

(A) Yeast two hybrid assay comparing the binding strength of R141Q and R141E to Sllbo (C/EBP). The R141Q mutation decreased Slbo binding while R141E bound Slbo as strongly than WT Trbl as demonstrated by yeast cells co-expressing Slbo prey. R141Q grows in the presence of up to 25mM 3AT growth inhibitor, while Trbl and R141E bait grow well in the presence of up to 100mM 3AT. Note that equal numbers of transformed yeast cells were used to seed the plates used for assay.

(B) Co-IP of Trbl from fat body extracts misexpressing Trbl or R141E and subjected to western blotting probed with pan-Akt shows R141E binds Akt more strongly than Trbl.

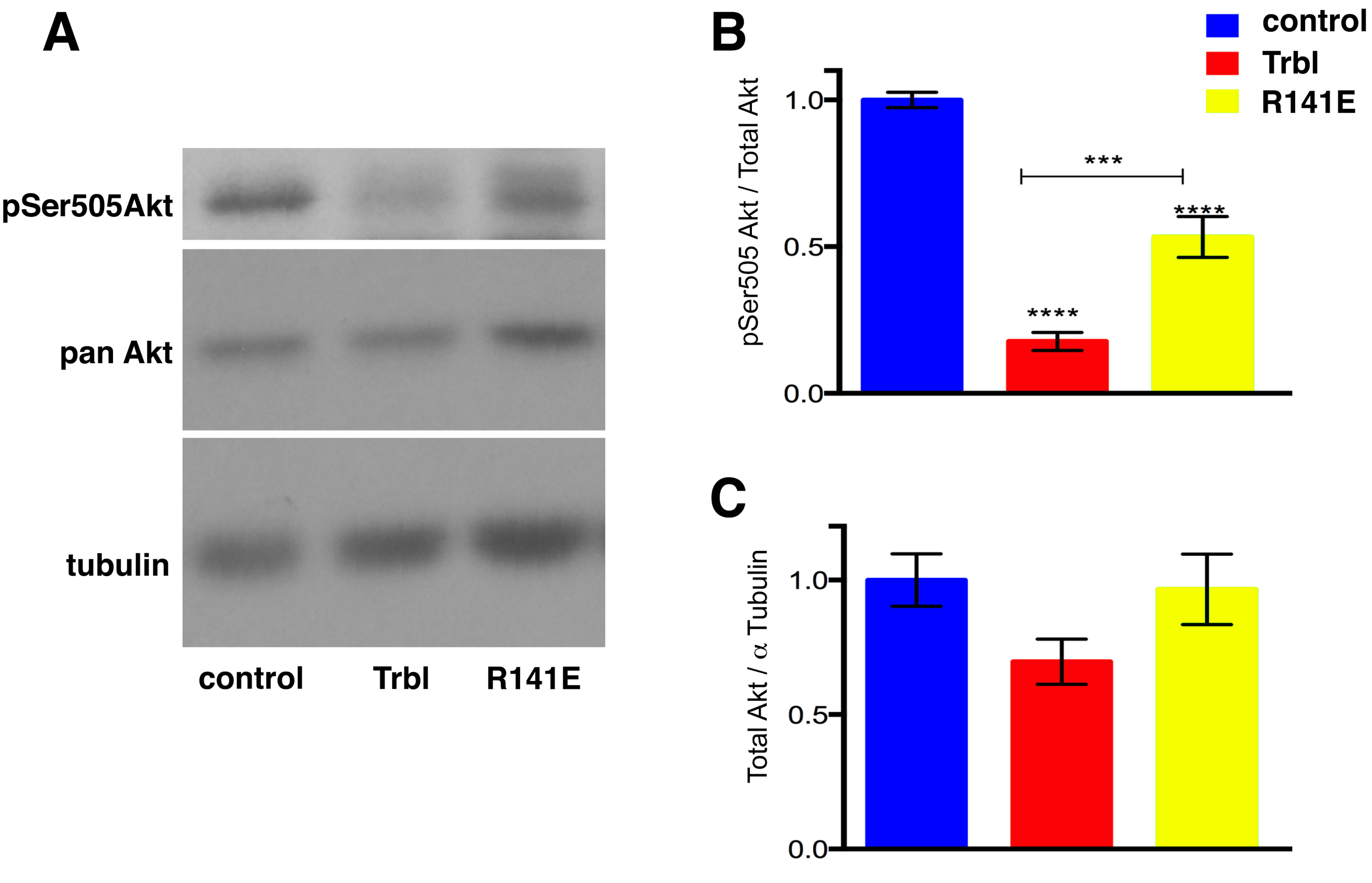

Disease Models & Mechanisms • Supplementary information

Figure S6. The R141E mutation compromises Trbl-mediated reduction in Akt activation

(A) Representative western blot of fat body extracts from age matched larvae driving transgene expression by *r4-GAL4* were probed with phospho-Akt, panAkt and β-tubulin antisera by stripping and re-probing the same blot to reveals that the R141E mutation blocks Akt phosphorylation less effectively than WT Trbl. Genotypes are: (1) control, *r4-GAL4*/+; (2) Trbl, *UAS-Trbl*; *r4-GAL4* (4) R141E, *UAS-Trbl*^{R141E}/*r4-GAL4*.

(B and C) Quantification of at least three independent western blots of fat body extracts show that Trbl decreases Akt activation and the R141E mutation blunts this effect (B); for misexpression of either, total Akt levels were unaffected (C). β-tubulin band was used as loading control and results were normalized to control. Genotypes are same as (A). Statistical analysis was performed with one-way ANOVA followed by Tukey post-hoc on Graph Pad Prism. Error bars represent mean ± SEM.

Disease Models & Mechanisms • Supplementary information

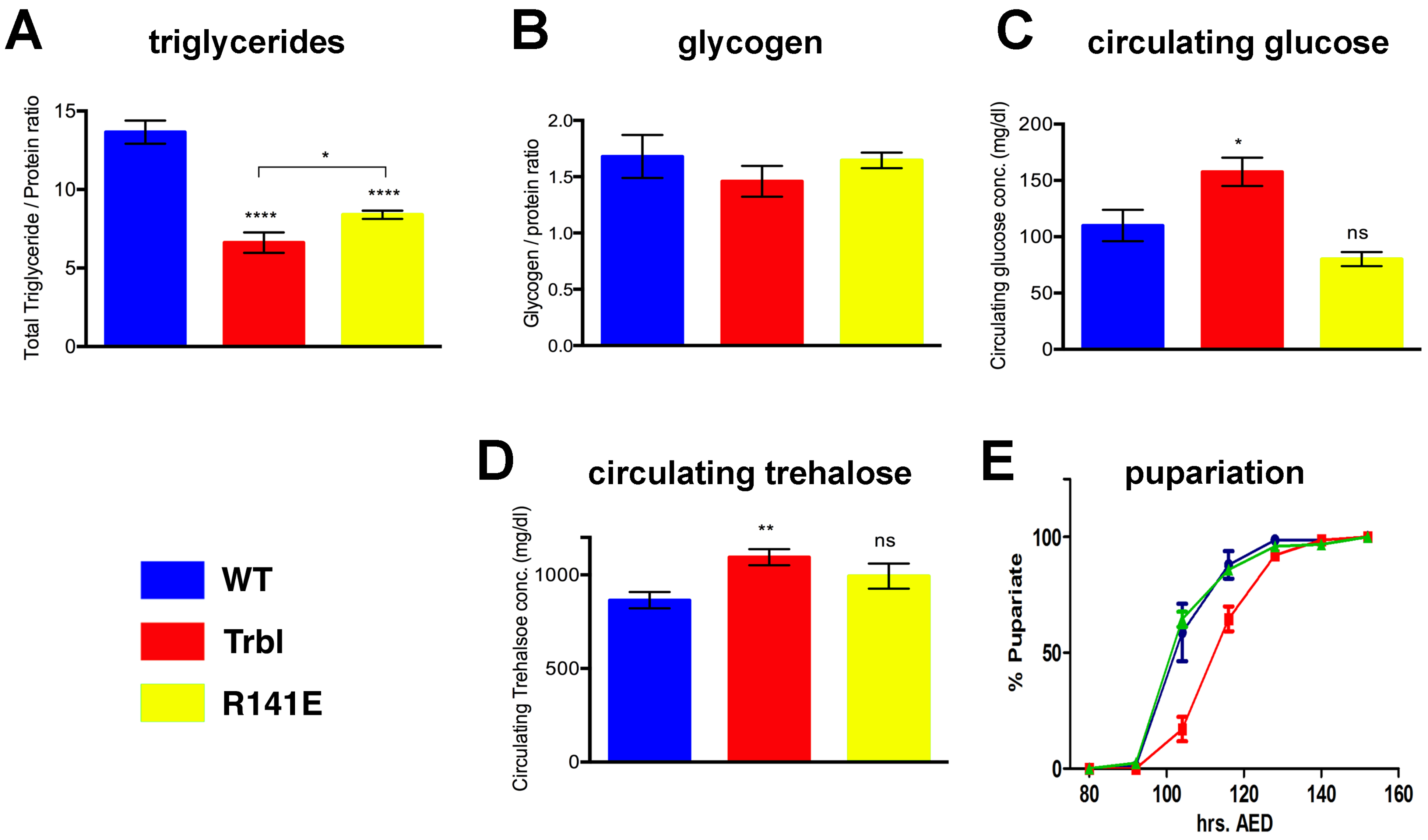

Disease Models & Mechanisms • Supplementary information

Figure S7. The R141E mutation compromises some Trbl-mediated effects on metabolite levels

(A-D) Assays for stored or circulating metabolites were performed on age-matched larvae and reveal the effect of misexpressing WT Trbl or R141E in the fat body. (A) stored triglyceride, (B) stored glycogen, (C) circulating glucose, (D) circulating trehalose. As before, triglyceride and glycogen contents are presented as relative to the protein content of the tissue sample to normalize the weight difference between control and transgene-expressing larva. (A-E), n=20. Statistical analysis was performed with one-way ANOVA followed by Tukey post-hoc on Graph Pad Prism. Error bars represent mean ± SEM.

(E) Compared to control, fat body misexpression of R141E delayed pupariation to a similar extent as WT Trbl. Experiment was done in biological triplicate. AED, after egg deposit. Genotypes are: (1) control, *r4-GAL4*/+; (2) Trbl, *UAS-Trbl*; *r4-GAL4* (3) R141E, *UAS-Trbl*^{R141E}/ *r4-GAL4*. All experiments were done in biological triplicate. Error bars represent Mean ± SEM.
